# Using WGCNA and transcriptome profiling to identify hub genes for salt stress tolerance in germinating soybean seeds

**DOI:** 10.3389/fpls.2025.1569565

**Published:** 2025-08-08

**Authors:** Lijun Pan, Yifan Chen, Zeyu Ren, Dalia Mohamedkheir Khojely, Siyu Wang, Yueming Li, Seifeldin Elrayah Ibrahim, Sujie Fan, Yang Song, Zhuo Zhang, Jian Wei

**Affiliations:** ^1^ Plant Biotechnology Center, College of Agronomy, Jilin Agriculture University, Changchun, Jilin, China; ^2^ Gezira Research Station, Agricultural Research Corporation (ARC), Wad Madani, Sudan

**Keywords:** soybean, germination, salt stress tolerance, transcriptome profiling, WGCNA

## Abstract

Salinized soil can significantly hinder soybean growth, leading to a reduction in overall yield. To address this issue, identifying key genes related to salt tolerance in soybeans is essential for improving their resistance to salinity and ensuring sustainable development of soybean production. While current research predominantly focuses on salt tolerance during the seedling stage, there is still a lack of comprehensive studies on the genes involved in salt tolerance during the germination stage. This study established the optimal screening criteria by phenotyping the salt-tolerant variety R063 and the salt-sensitive variety W82 during the germination stage under salt stress. RNA-seq analysis was performed on 24 samples from both varieties at 36 and 48 hours under two different salt concentrations (0 and 150 mM/L NaCl). Differential expression analysis revealed that the salt-tolerant variety R063 exhibited the fewest differentially expressed genes (DEGs) compared to its control after 48 hours of salt stress. A total of 305 DEGs were commonly identified between the salt-tolerant variety R063 and the salt-sensitive variety W82 under salt stress at both time points. Additionally, 187 DEGs were commonly identified between R063 under salt stress and its corresponding control group across the two time points. Gene ontology (GO) enrichment analysis revealed that the differentially expressed genes were significantly enriched in ADP binding, monooxygenase activity, oxidoreductase activity, defense response, and protein phosphorylation signaling pathways. The weighted gene co-expression network analysis (WGCNA) method was employed to identify modules strongly correlated with salt tolerance during soybean germination. Candidate genes associated with soybean sprouting salt tolerance were identified by evaluating the connectivity and expression profiles of genes within these modules. These findings provide a theoretical foundation for further elucidating the molecular mechanisms underlying salt tolerance during soybean germination and present new genetic resources for studying this trait.

## Introduction

According to incomplete statistics from UNESCO and the Food and Agriculture Organization of the United Nations (FAO, 2024), saline soils cover 1,381 million hectares, accounting for 10.7% of the world’s total land area. In China, the area of saline-alkaline land is approximately 3.6 × 10^7^ hectares, accounting for 4.88% of the country’s total available land resources (Li et al., 2014). Soda saline-alkaline land in the western part of the Songnen Plain in northeastern China represents one of the world’s three largest concentrations of soda saline-alkaline soils (Wei et al., 2020). Soil salinization not only restricts the expected growth of plants but also significantly impacts the development of China’s national economy. Screening and breeding salt-tolerant crop varieties is one of the most effective strategies to mitigate the inhibitory effects of saline-alkaline soils on plant growth (Zou et al., 2022).

To cope with these stresses, plants have developed complex adaptive mechanisms through long-term evolutionary processes. While extensive research has been conducted on salt stress tolerance in soybeans, most studies have focused on tolerance during later stages of growth, with fewer investigations addressing salt tolerance during the germination stage. Ma et al. (2021) found that overexpression of the gene GmNFYA13 improved salt tolerance in transgenic soybean plants, while Li et al. (2017) reported that overexpression of the gene GmFDL19 enhanced salt tolerance in soybean seedlings. Hu et al. (2022) conducted transcriptome sequencing of both salt-tolerant and salt-sensitive soybean varieties during the seedling stage at three time points, identifying 6644 differentially expressed genes. Enrichment analysis revealed that these genes were involved in phytohormone signaling, redox processes, phenylpropanoid biosynthesis, mitogen-activated protein kinase (MAPK) signaling, and ribosome metabolism, all of which may play key roles in salt stress response. Additionally, through interlocking localization and genome-wide association studies, Zhang et al. (2019) identified the major QTL qST-8, which was significantly associated with salt tolerance during soybean seed germination.

Previous studies have shown that the salt tolerance mechanisms during germination and the corresponding genes differ from those identified at later growth stages. Additionally, the number of studies and reference identifications related to salt tolerance at the germination stage is limited, and the evaluation criteria for this stage do not fully align with those for other growth periods. Therefore, further investigation into the salt tolerance mechanisms during germination is required. For example, GmSALT3, a salt tolerance gene identified during the seedling stage, is unrelated to salt tolerance during the emergence stage (Liu et al., 2016). The focus on mining salt tolerance genes only during the seedling stage does not lead to a comprehensive improvement in soybean’s overall salt tolerance. If seeds lack salt tolerance during germination, the seedling emergence rate will be reduced, which can seriously affect soybean cultivation in saline soils.

The use of transcriptome sequencing technology is increasingly prevalent in the identification of plant salt tolerance genes. This technology enables more precise qualitative and quantitative analysis of the types and abundance of genes expressed in specific cells or tissues under defined conditions. It has been widely applied in crop research for gene mining and the exploration of mechanisms such as disease resistance and stress tolerance, providing novel insights and methodologies for functional genomics studies across different organisms (Imadi et al., 2015; Afzal et al., 2020). While transcriptome sequencing has become a well-established tool for gene discovery, fewer studies have integrated this technique with others to identify pivotal genes. WGCNA (Weighted Gene Co-expression Network Analysis) is a systems biology approach that utilizes transcriptomic sequencing data to construct gene co-expression networks. It primarily involves grouping genes with similar expression patterns into modules, building co-expression networks, and identifying key hub genes. WGCNA has been successfully applied to identify genes in rice (Wang et al., 2022), wheat (Luo et al., 2019), and maize (Ma et al., 2021). Yao et al. (2023)employed transcriptomics and WGCNA analyses to identify hub genes regulating seed size and oil content during the developmental stages of both wild and cultivated soybeans. Similarly, Tang et al. (2023) utilized transcriptomic and WGCNA analyses to uncover the involvement of hub genes related to drought stress in the tillering seedlings of sugarcane.

**Figure 1:**
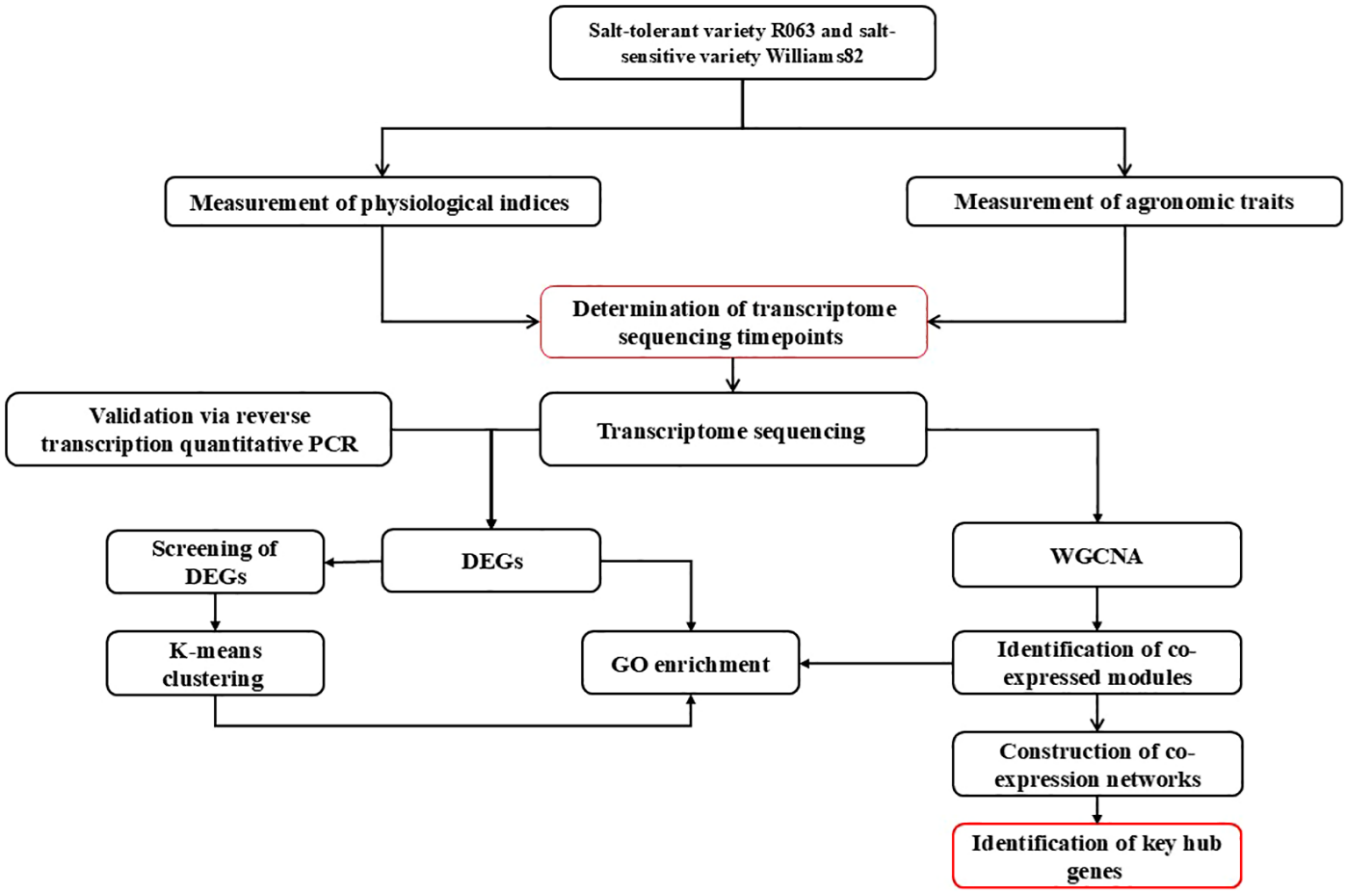
Technology roadmap. The workflow investigates salt stress responses in two rice varieties (salt-tolerant R063 and salt-sensitive Williams 82) through a multi-step approach. Key stages include:**(1)** Physiological and Agronomic Assessments: Measuring indices and traits to identify critical timepoints for transcriptomic analysis. **(2)** Transcriptome Profiling: RNA-seq identifies differentially expressed genes (DEGs), followed by GO enrichment and WGCNA to detect co-expressed gene modules. **(3)** Validation and Network Analysis: RT-qPCR confirms RNA-seq results, while co-expression networks highlight hub genes central to salt tolerance. **(4)** Integration of Data: Combining physiological, transcriptomic, and network analyses to pinpoint key regulatory genes, providing molecular insights for crop improvement under salinity stress.

As one of the major food crops in China, soybean consumption has been steadily increasing in recent years (He et al., 2019). Seed germination represents a critical developmental stage in the life cycle of soybeans (Zhang et al., 2021), and abiotic stresses encountered during seed germination can slow down soybean seed germination, reduce germination rate, or lead to seed rot and death, resulting in large-scale seedling shortages and crop failure and causing significant economic losses to soybean production (Liu et al., 2020). Screening salt-tolerant soybean varieties and mining salt-tolerant genes are of great significance to the development and utilization of soybean germplasm resources. In this study, we used transcriptome and WGCNA technologies to study soybean seeds at the germination stage after salt stress. K-means clustering analysis and gene ontology (GO) enrichment analysis were performed for differential genes co-existing between salt-tolerant varieties and their controls at 2-time points and for differentially expressed genes (DEGs) co-existing between salt-tolerant and salt-sensitive varieties at 2-time points, respectively, and co-expression analyses were performed to identify the pivotal genes involved in salt stress tolerance in soybean during the germination period (**Figure 1**). This study provides a new perspective on understanding.

This study has the following contributions:

- By analyzing the transcriptome data of salt-tolerant and salt-sensitive varieties of germinating soybeans, key genes related to salt tolerance were identified.
- By combining high-throughput transcriptome data and WGCNA analysis, it was possible to systematically identify hub genes that play key roles in the process of salt stress tolerance during the germination period and which play central roles in regulating the response to salt stress.
- WGCNA can help to reveal the co-expression patterns among genes, and further provide a theoretical basis for understanding the salt tolerance mechanism and improving the salt resistance of soybean.

## Materials and methods

The “Plant Materials and Stress Treatment” subsection provides the foundation for the experiment, detailing the types of soybeans used, the treatment conditions, and the phenotyping approach. The “Water Absorption Rate”, “SOD Activity”, “POD Activity” and “MDA content” subsections focus on physiological measurements that assess the plants’ responses to salt stress, providing crucial data on the impact of salt stress at the biochemical level. RNA sequencing and WGCNA provide molecular insights into gene expression changes and network analysis, which are further validated through RT-qPCR. This multi-level approach—spanning phenotypic, physiological, biochemical, and molecular analyses—ensures a robust and comprehensive study of salt stress tolerance in soybeans.

### Plant materials and stress treatment

Salt-tolerant soybean variety ‘R063’ as well as salt-sensitive soybean variety ‘W82’ were used in this study. Fifty soybean seeds were selected and placed flat in petri dishes with three replications in each group. The soybean seeds were sterilized with alcohol for 30 seconds and then washed with distilled water three times. The dark culture was incubated for two days and then shifted to the light culture environment. Seeds were subjected to salt stress treatments at varying concentrations (0, 100, 150, and 200 mM NaCl) to assess germination responses. Seed mass was measured at 12-hour intervals for a total of 72 hours. To maintain experimental conditions, water was replenished and filter paper was replaced every 24 hours, as described by Kan et al. (2015). The optimal salt concentration (150 mM NaCl) was determined through phenotypic screening of germination traits.

### Imbibition rate

Select 10 seeds of uniform size for each sample, with three replicates per group. Initially, After absorbing water, soybean seeds were placed in an oven at 105°C, inactivated for 30 min, and then dried at 80°C to constant weight as ${\text{P}}_{1}$ . After soaking, gently blot the seeds with filter paper or cloth to remove excess surface moisture, then weigh the soaked seeds and record this total mass as ${\text{P}}_{2}$ (Smith et al., 1961). The formula to calculate the imbibition rate is given by:


$$\text{Imbibition Rate }\left(\%\right)=\frac{P_{2}-P_{1}}{P_{1}}\times 100\%$$

. P_1_ is the dry weight of the seed, ${\text{P}}_{2}$ is the total weight of the seed after imbibition.

### Superoxide dismutase activity

The activity of Superoxide Dismutase (SOD) was measured using the nitro blue tetrazolium (NBT) method (Fridovich, 1986). The reaction mixture consisted of 1.5 mL of 0.05 mol/L phosphate buffer, 0.3 mL of 130 mM methionine (Met) solution, 0.3 mL of 750 μM NBT solution, 0.3 mL of 100 μM EDTA-Na2, 0.3 mL of 20 μM riboflavin, and 0.05 mL of enzyme extract, making up a total reaction volume of 3 mL. The tubes containing the reaction mixture were incubated under a light intensity of 4000 lux at 25°C for 30 minutes. One unit of SOD activity is defined as the amount of enzyme required to inhibit the reduction rate of NBT by 50% under assay conditions. SOD activity can be calculated using the following formula:


$$\text{Total SOD Activity}=\frac{(C_{0}-C_{S})\times V_{T}}{\left(C_{0}\times 0.5\times F_{W}\times V_{1}\right)}$$

. ${\text{C}}_{0}$ is the absorbance of the light control (the tube exposed to light). ${\text{C}}_{S}$ is the absorbance of the sample. ${\text{V}}_{T}$ is the total volume of the diluted sample (mL). ${\text{V}}_{1}$ is the sample used for measurement (mL). ${\text{F}}_{W}$ is the fresh weight of the sample (g). The total SOD activity is expressed in enzyme units per gram of fresh weight. Protein concentration is given as milligrams of protein per gram of fresh weight (mg/g).

### Peroxidase activity assay using Guaiacol method

Determination of POD activity (Guaiacol method) (Gao et al., 2021).

1. Reagent Preparation: 100 mmol/L Phosphate Buffer Solution (PBS, pH 6.0): Prepare by mixing 61.5 mL of 1 M sodium phosphate dibasic (Na_2_HPO_4_) (Solution A) with 438.5 mL of 1 M sodium phosphate monobasic (NaH_2_PO_4_) (Solution B), then dilute to 1,000 mL with deionized water. The final pH is adjusted to 6.0 using a calibrated pH meter.
2. Reaction Mixture Preparation: Combine 50 mL of PBS (100 mmol/L, pH 6.0) with 28 μL of guaiacol (2-methoxyphenol) in a beaker. Heat gently with continuous stirring to dissolve the guaiacol. After cooling, add 19 μL of 30% (v/v) hydrogen peroxide (H_2_O_2_) and mix thoroughly. Store the solution at 4°C for subsequent use.
3. Sample Extraction: Accurately weigh 1.0 g of germinating soybean seeds and homogenize them in a pre-cooled mortar using an appropriate volume of PBS (pH 6.0). Centrifuge the homogenate at 4,000 × g for 15 min at 4°C. Transfer the supernatant to a 100 mL volumetric flask and dilute to the mark with PBS. Store the enzyme extract at 4°C until analysis.

Spectrophotometric Measurement: For each sample, prepare three test tubes: Blank Tube: Add 3.0 mL of reaction mixture and 1.0 mL of PBS. Test Tubes: Add 3.0 mL of reaction mixture and 1.0 mL of enzyme extract. Immediately initiate the reaction by adding the enzyme solution and record the absorbance at 470 nm at 30 s intervals for 5–10 measurements. Perform measurements sequentially to avoid interference from simultaneous reactions.

Enzyme Activity Calculation: POD activity is expressed as the change in optical density (ΔOD_470_) per minute, normalized to a standard unit. One unit (U) of POD activity is defined as the amount of enzyme required to catalyze the oxidation of guaiacol, resulting in a ΔOD_470_ of 0.01 per minute under the specified assay conditions. The activity is calculated using the formula:


$$\text{POD Activity }[\text{U}/(\text{g}\cdot \text{min})]=\left(\Delta {\text{A}}_{470}\times 100\times \text{Vt}\right)/\left(\text{W}\times \text{Vs}\times \text{t}\right)$$

. In the equation, ΔA_470_ represents the change in absorbance during the reaction period; W denotes the fresh weight (g) of the sample; t indicates the reaction time (min); Vt refers to the total volume of the enzyme extraction solution; and Vs corresponds to the volume of enzyme solution used during measurement.

### Malondialdehyde content assay

MDA content was measured using a commercial MDA assay kit (Sangon Biotech, Shanghai, China).

(1) Reagent Addition and Reaction Protocol

The reaction was performed in two tubes: a test tube (containing the sample) and a blank tube (without the sample). Reagent volumes were as follows: MDA detection working solution: 600 μL (test and blank tubes); Sample supernatant: 200 μL (test tube only); Distilled water: 200 μL (blank tube only); Reagent Three: 200 μL (both test and blank tubes).

The mixture was incubated in a 100 °C water bath for 60 minutes (with lids tightened to prevent evaporation) and then cooled rapidly in an ice bath. After cooling, the samples were centrifuged at 10,000 × g for 10 minutes at room temperature, and the supernatant was transferred to 1 mL quartz cuvettes for absorbance measurement.

(2) Spectrophotometric Analysis and Calculation

Absorbance values were measured using a visible spectrophotometer at 532 nm and 600 nm. The corrected absorbance (ΔA) was calculated as follows:


$$\Delta {\text{A}}_{532}={\text{A}}_{532a}-{\text{A}}_{532b}$$

$$\Delta {\text{A}}_{600}={\text{A}}_{600a}-{\text{A}}_{600b}$$

$$\Delta \text{A}=\Delta {\text{A}}_{532}-\Delta {\text{A}}_{600}$$

A_532a,_ A_600a_: Absorbance values of the test tube at 532 nm and 600 nm, respectively; A_532b,_ A_600b_: Absorbance values of the blank tube at 532 nm and 600 nm, respectively.

The MDA content (nmol/g mass) was quantified using the formula:


$$\text{MDA}\;\text{Content}\;\text{(nmol}/\text{g)}=[\Delta \text{A}\times {\text{V}}_{1}÷(\epsilon \times \text{d)}\times 10^{9}]/(\text{W}\times {\text{V}}_{2}÷{\text{V}}_{3})$$

The variables in the formula are defined as follows: V_1_: Total reaction volume, fixed at 1.0 × 10⁻³ L; ***ϵ***: The MDA molar absorption coefficient, 1.55 × 10^5^L/mol/cm; V_2_: Sample volume added, 0.2 ML. d: Cuvette light path diameter, 1 cm; V_3_: Volume of extract added, 1 ml; Cpr: Sample protein concentration, mg/L. W: Sample weight, g.

### RNA sequencing

Transcriptome analysis was performed on 36-h and 48-h samples of salt-tolerant versus salt-sensitive seeds. Each treatment consisted of three replicates, yielding a total of 24 samples for RNA sequencing. Trizol reagent (Invitrogen, USA) was used to isolate total RNA from the samples. DNase I was used to remove contaminating genomic DNA from the RNA.RNA concentration and purity were detected using the UV spectrophotometric NanoDrop (NanoDropND-1000 UV-Vis spectrophotometer) (Smith et al., 1961). Qualified RNA was processed for RNA sequencing to construct RNA-seq libraries, which was performed by Biomarker Technologies Co., Ltd. (Beijing, China) on the Illumina sequencing platform. The raw image data generated by sequencing was converted from base detection to sequence data, called raw data/raw reads. The raw data from sequencing was quality assessed by FastQC, quality clipped by Trimmomatic to obtain relatively accurate valid data, and the valid data from the samples were aligned to the reference genome using HISAT2. The Glycine max cv. Williams 82 reference genome (Wm82.a2.v1) and corresponding gene annotation files were obtained from SoyBase [https://www.soybase.org/dlpages/index.php], a curated soybean genomic resource. Genes with |log_2_ fold-change| ≥ 1 and FDR< 0.05 were considered differentially expressed. GO enrichment analysis of differentially expressed genes was performed using the Database for Annotation, Visualization, and Integrated Discovery (DAVID) (Sherman et al., 2022). Selection pathway enriched to differential genes greater than 6 and less than 1500, and all false discovery rate (FDR) adjusted p-values (q-values) with q< 0.05 were considered significant (Jhan et al., 2023).

### WGCNA

WGCNA was performed on the transcriptome data using R software version 4.2.2 and the WGCNA package (Langfelder and Horvath, 2008; Wang et al., 2023). The soft threshold was determined based on the scale-free network distribution principle. The threshold parameter β for creating the adjacency matrix was selected when the fit curve first approached 0.8 (Zhao et al., 2024). Set the soft threshold to β = 15 to realize a scale-free network. The adjacency matrix was transformed into a Topological Overlap Matrix (TOM) using the TOM similarity function, thereby constructing the gene connectivity network (Dileo et al., 2011). Finally, gene modules were generated based on high gene significance (GS, the association between gene expression and traits) and module membership (MM, the correlation between gene expression and module eigengenes) (Azam et al., 2023). These gene modules were clustered using the dynamic tree-cut method, and the co-expression networks were visualized using Cytoscape version 3.10.0 (Shannon et al., 2003). Chromosomal localization of hub genes using TBtools in R (Chen and Xia, 2022).

### Quantitative real-time PCR verification

RNA was extracted from salt-tolerant and salt-sensitive varieties treated with zero mM and 150 mM NaCl at 36 hours and 48 hours post-treatment using Trizol and then reverse-transcribed into cDNA. Specific quantitative primers were designed using Primer Premier 5 software (**Supplementary Table S1**) for RT-qPCR reactions, with *Glyma.12G051100* as the reference gene. Each sample included three biological replicates. The relative expression levels of the genes were calculated using the 2^-ΔΔCt^ method (Amorim et al., 2018), and graphs were generated using GraphPad Prism 8 software.

### Statistical analysis

All data were analyzed using one-way analysis of variance (ANOVA) to assess significant differences among treatments and time points. Duncan’s multiple range test was applied for *post hoc* comparisons at a significance level of p< 0.05. All statistical analyses and visualizations were performed using GraphPad Prism 8. Data are presented as means ± standard error (n = 3).

## Results

### Phenotypic response of soybean seeds to salt stress

**Figure 2:**
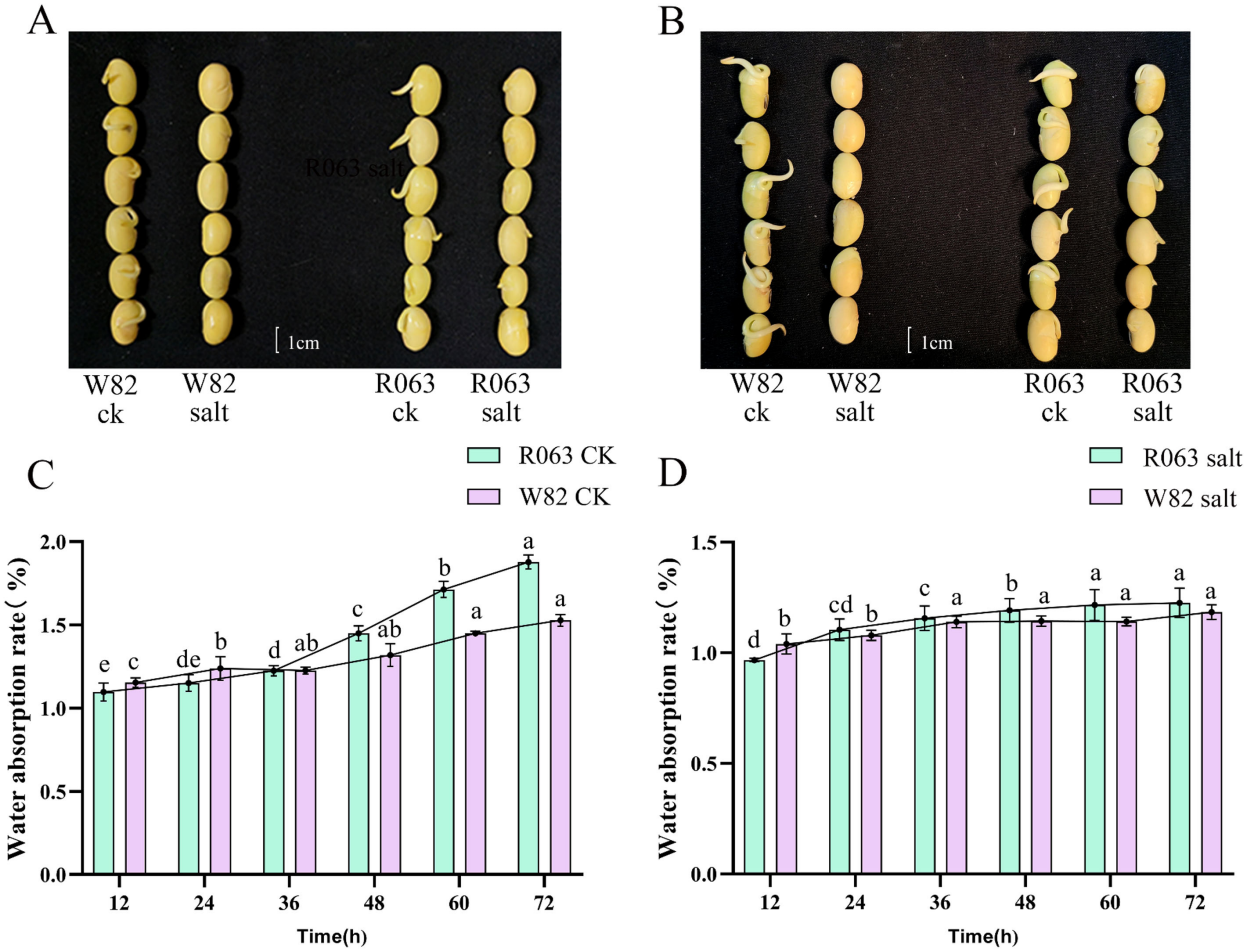
The critical stages of soybean seed response to salt stress at 36h and 48h. **(A)** Seed phenotypes of W82 (left) and R063 (right) varieties under control conditions and salt stress at 36 hours. **(B)** Seed phenotypes of W82 (left) and R063 (right) varieties under control conditions and salt stress at 48 hours. **(C)** Dynamic changes in water absorption rates over time for the control groups of W82 and R063 soybean varieties. **(D)** Dynamic changes in water absorption rates over time for W82 and R063 soybean varieties under salt stress. Scale bar, 1 cm. Data are presented as means ± standard error (n = 3). Different lowercase letters indicate statistically significant differences among time points within the same treatment group at p< 0.05, as determined by one-way ANOVA followed by Duncan’s multiple range test.

Taking R063 and W82 as materials, the seeds were treated with 150 mmol/L NaCl for three days, respectively, during the seed germination period; with the increase of salt stress time, the germination rate of the two varieties appeared to be significantly changed, and it was observed that the seeds showed significant changes in germination rate between 36 h and 48 h, and the germination rate of W82 was affected by the salt stress more than the germination rate of R063 was affected seriously (**Figures 2A, B**). Analysis of water absorption rates revealed that over time, the rate for R063 gradually increased, whereas that for W82 showed a gradual decline. This indicates that salt stress significantly restricted the water absorption rate of W82 but had a lesser effect on R063 (**Figures 2C, D**).

### Analysis of SOD activity

**Figure 3:**
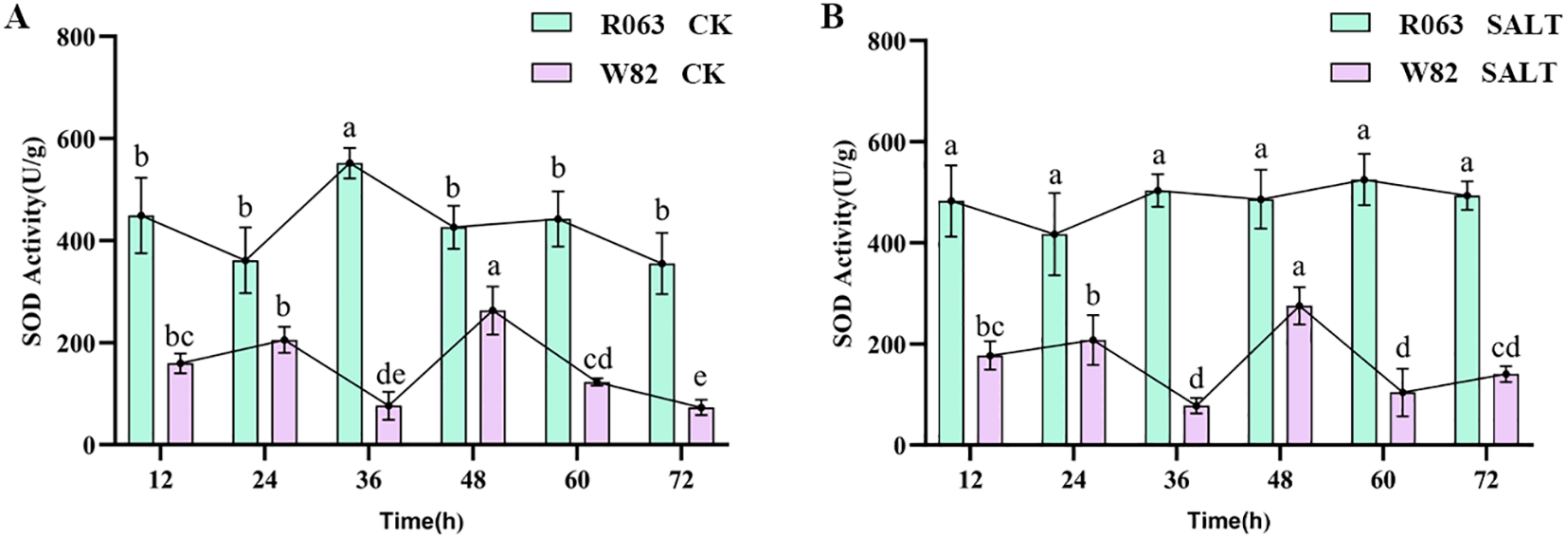
Changes in SOD activity during germination in soybean with different tolerances. **(A)** Changes in SOD activity of W82 and R063 at six time points under normal conditions. **(B)** Changes in SOD activity at six time points under salt stress conditions in W82 and R063. Data are presented as means ± standard error (n = 3). Different lowercase letters indicate statistically significant differences among time points within the same treatment group at p< 0.05, as determined by one-way ANOVA followed by Duncan’s multiple range test.

SOD is a key antioxidant enzyme for plants to cope with oxidative stress, which reduces the accumulation of reactive oxygen species (ROS) and mitigates the oxidative damage of salt stress on the cell membrane system by catalyzing the conversion of superoxide anion radicals (O_2_⁻) to H_2_O_2_ and O_2_. In this study, salt-tolerant variety R063 and salt-sensitive variety W82 were subjected to salt stress treatment (150 mmol/L NaCl) by hydroponics, and the samples were collected at six time points—12, 24, 36, 48, 60, and 72 h, respectively—and the SOD activity was determined using the nitrogen blue tetrazolium (NBT) photoreduction method. The results showed that under normal conditions, the SOD activity of R063 was significantly higher than that of W82 and reached a peak at 36 h and then stabilized, while the SOD activity of W82 reached a peak at 48 h and then gradually decreased. Under salt stress, the SOD activity of R063 was significantly higher than that of W82, and its trend was consistent with that of the control, while the activity of W82 fluctuated greatly after salt stress. This result indicated that R063 effectively alleviated salt stress-induced ROS accumulation by maintaining a higher and stable SOD activity (**Figures 3A, B**).

### Analysis of POD activity

**Figure 4:**
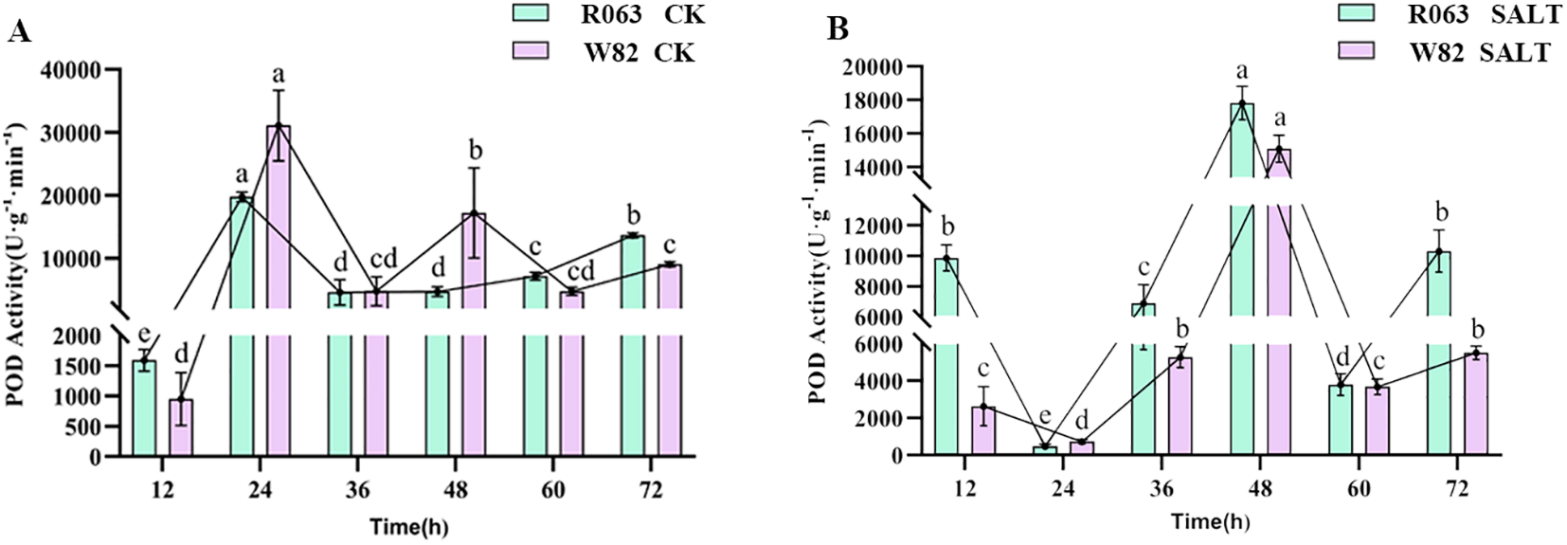
Changes in POD activity during germination in soybean with different tolerances. **(A)** Changes in POD activity of W82 and R063 at six time points under normal conditions. **(B)** Changes in POD activity at six time points under salt stress conditions in W82 and R063. Data are presented as means ± standard error (n = 3). Different lowercase letters indicate statistically significant differences among time points within the same treatment group at p< 0.05, as determined by one-way ANOVA followed by Duncan’s multiple range test.

POD is an important enzyme for H_2_O_2_ scavenging in plants, and changes in its activity can reflect the intensity of plant response to oxidative stress. The results of this study showed that the POD activities of both R063 and W82 peaked at 24 h under normal conditions. Under salt stress, the POD activities of R063 and W82 had the lowest activity at 24 h and the peak activity at 48 h. The POD activities of R063 and W82 were also found to be the lowest at 24 h and the peak activity at 48 h (**Figures 4A, B**).

### Analysis of MDA content

**Figure 5:**
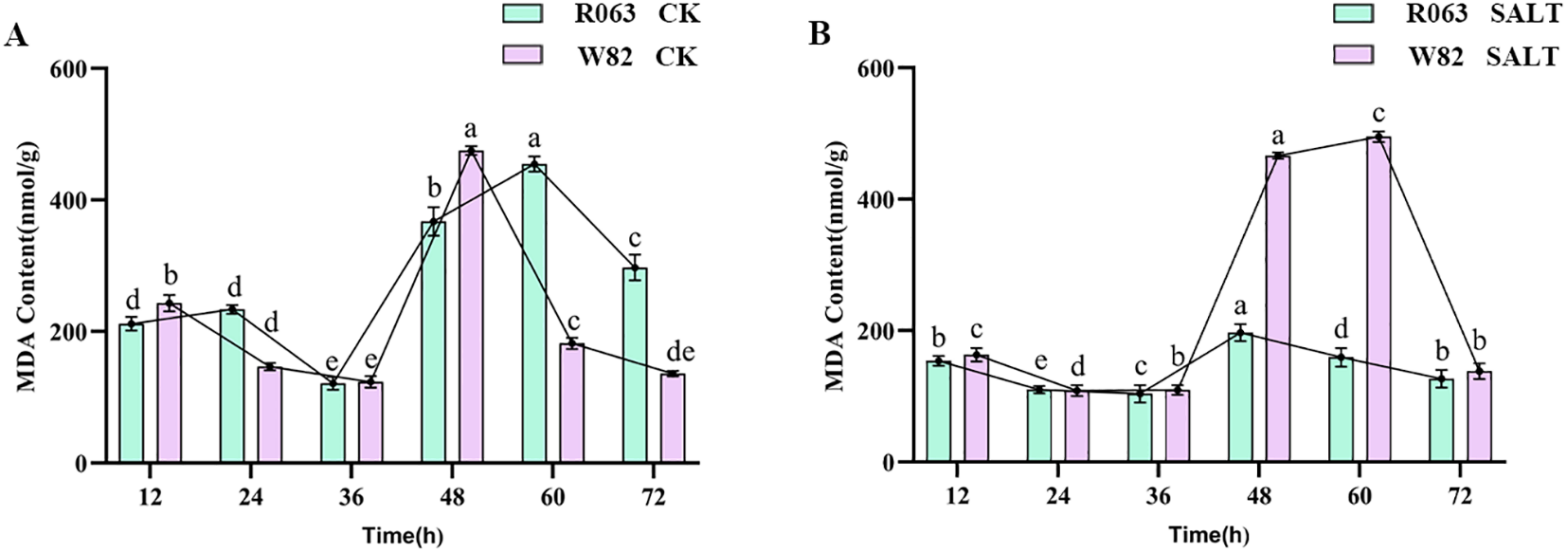
Changes in MDA content during germination of different tolerant soybeans. **(A)** Changes in MDA content of W82 and R063 at six time points under normal conditions. **(B)** Changes in MDA content at six time points under salt stress conditions in W82 and R063. Data are presented as means ± standard error (n = 3). Different lowercase letters indicate statistically significant differences among time points within the same treatment group at *p*< 0.05, as determined by one-way ANOVA followed by Duncan’s multiple range test.

MDA is a hallmark product of membrane lipid peroxidation, and its content can reflect the degree of cell membrane damage. The results of this study showed that, under normal conditions, the MDA content of R063 showed a tendency to increase and then decrease from 12 to 36 h, and then increased again and then decreased from 48 to 72 h. The MDA content of W82 decreased continuously from 12 to 36 h, but increased significantly after 36 h. The MDA content of G68 was significantly lower than that of Williams82 under salt stress, and its fluctuation was lower than that of Williams82. Under salt stress, the MDA content of R063 was significantly lower than that of W82, and the fluctuation of MDA content was small, indicating that the degree of membrane lipid peroxidation was low (**Figures 5A, B**).

### Comprehensive analysis of physiological indicators and selection of transcriptome sequencing time points

Based on a detailed analysis of antioxidant enzyme activity under salt stress, we selected 36 hours and 48 hours as the key time points for transcriptome sequencing. Although POD activity peaked at 24 hours under control conditions, its trend differed markedly under salt stress. Specifically, POD activity was lowest at 24 hours and increased substantially afterward, reaching a peak at 48 hours in both the salt-tolerant (R063) and salt-sensitive (W82) varieties. This delayed response suggests that the activation of peroxidase-dependent antioxidant defense mechanisms occurs later during salt stress.

In contrast, SOD activity showed the largest divergence between R063 and W82 at 36 hours, reflecting early-stage antioxidant activation. At 48 hours, the increase in MDA content in W82 indicated substantial oxidative damage, while POD activity reached its maximum, this suggests that a stress-induced enhancement of peroxidative enzyme activity serving to restore redox homeostasis. Therefore, 36 hours represents the initiation of the stress response, and 48 hours reflects the transition to stress adaptation or damage control. These two stages provide meaningful insight into the physiological and molecular mechanisms of salt tolerance.

In summary, 36 h and 48 h were selected for subsequent transcriptome sequencing, which provided key data support for revealing the molecular mechanism of salt tolerance and the mining of resistance genes in soybean during the germination period.

### Transcriptome data analysis and quality assessment

Transcriptome sequencing was performed on 24 cDNA libraries constructed from R063 and W82 varieties and treated with 0 and 150 mmol/L NaCl at two time points (36 and 48 h). After filtering the 36-hour sequencing data, a total of 480,730,516 clean reads were obtained, with a Q30 ratio exceeding 95.81%, a GC content greater than 44.42%, and an overall alignment rate greater than 98% (**Supplementary Table S2**). Similarly, after filtering the 48-hour sequencing data, a total of 457,842,976 clean reads were obtained, with a Q30 ratio exceeding 95.82%, a GC content greater than 44.67%, and an overall alignment rate exceeding 98%. These results confirm that the transcriptome data were of high quality and suitable for downstream analysis (**Supplementary Table S3**). Group B corresponds to the 36-hour salt-treated Williams82 group, Group C represents the 36-hour control R063 group, and Group D is the 36-hour salt-treated R063 group (**Supplementary Table S4**). Group F corresponds to the 48-hour salt-treated Williams82 group, Group G represents the 48-hour control R063 group, and Group H is the 48-hour salt-treated R063 group (**Supplementary Table S5**).

### Differential expression gene analysis

**Figure 6:**
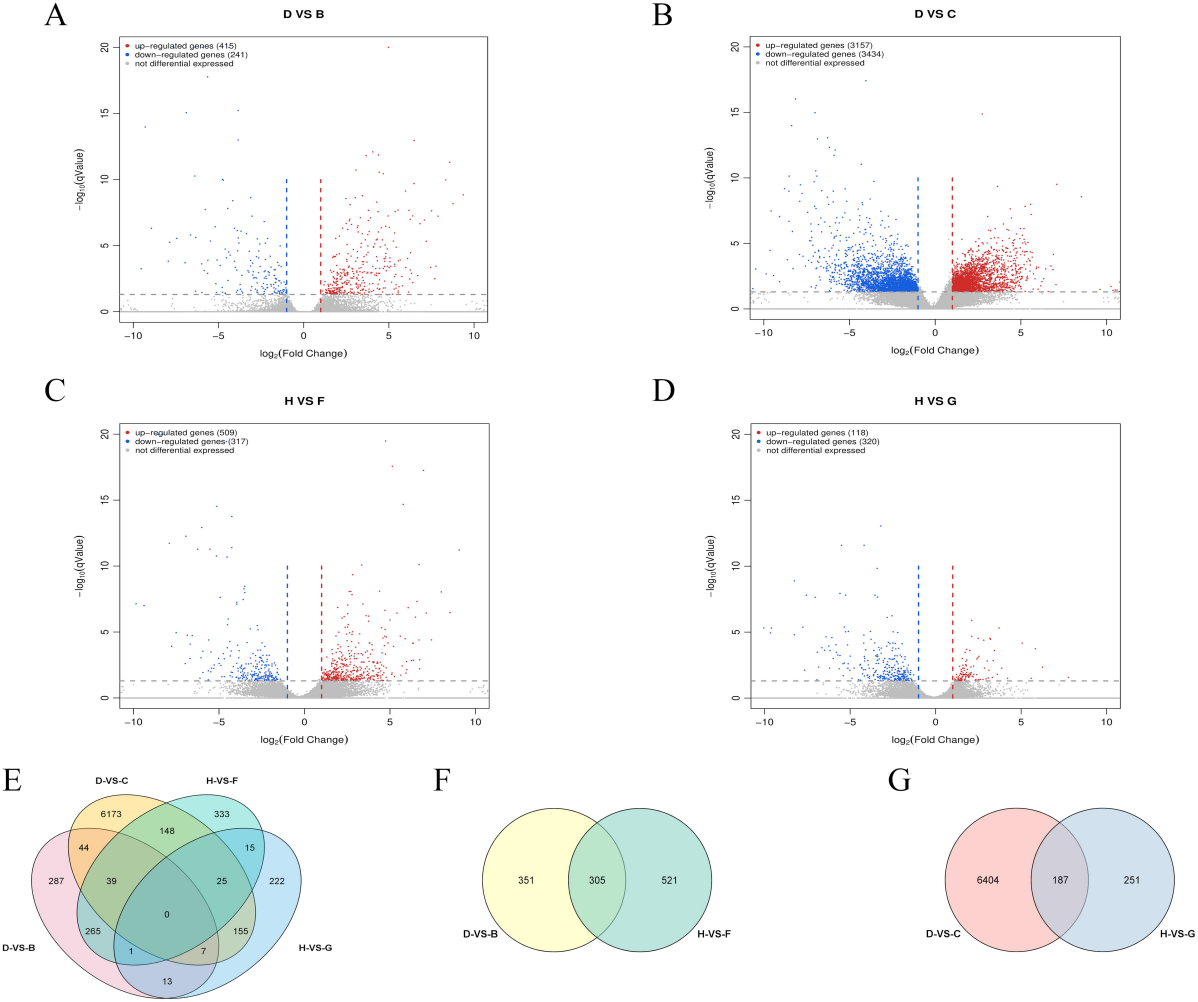
**RNA-seq analysis of W82 and R063 under salt stress at 36h and 48h stages**. **(A)** Volcano plot of DEGs for D-VS-B. **(B)** Volcano plot of DEGs for D-VS-C. **(C)** Volcano plot of DEGs for H-VS-F. **(D)** Volcano plot of DEGs for H-VS-G. **(E)** Venn diagram showing the overlap of DEGs among the four comparison groups. **(F)** Venn diagram of DEGs shared between 36 h and 48 h salt stress treatments in R063, relative to controls. **(G)** Venn diagram of DEGs shared between salt-tolerant and salt-sensitive genotypes under salt stress at 36 h and 48 h.

To investigate the regulatory mechanisms of salt-tolerant genes during soybean seed germination, pairwise comparisons were conducted among four cDNA libraries: D-VS-C, D-VS-B, H-VS-G, and H-VS-F. The groups were defined as follows: B group: W82 treated with salt at 36 hours; C group: R063 control at 36 hours; D group: R063 treated with salt at 36 hours; F group: W82 treated with salt at 48 hours; G group: R063 control at 48 hours; H group: R063 treated with salt at 48 hours. The comparison results revealed the following: D-VS-C identified 6,591 DEGs, including 3,157 upregulated and 3,434 downregulated genes (**Figure 6B**). D-VS-B identified 656 DEGs, with 415 upregulated and 241 downregulated genes (**Figure 6A**). H-VS-G showed 438 DEGs, comprising 118 upregulated and 320 downregulated genes (**Figure 6D**). H-VS-F exhibited 826 DEGs, including 509 upregulated and 317 downregulated genes (**Figure 6C**).

DC refers to the DEGs identified between the salt-tolerant soybean variety R063 under salt stress and its corresponding control group at 36 hours. HG refers to the DEGs identified between R063 under salt stress and its control group at 48 hours. DC-VS-HG represents the common DEGs identified between the salt-stressed R063 and its control group at both 36 and 48 hours. DB refers to the DEGs identified between the salt-tolerant variety R063 and the salt-sensitive variety W82 under salt stress at 36 hours. HF refers to the DEGs identified between R063 and W82 under salt stress at 48 hours. DB-VS-HF denotes the DEGs commonly identified between R063 and W82 under salt stress at both 36 and 48 hours. DC-VS-HG identified 187 DEGs (**Figure 6G**), with 47 upregulated and 139 downregulated genes across both time points. Notably, *Glyma.17G041200* was upregulated at 36 hours but downregulated at 48 hours. DB-VS-HF identified 305 DEGs (**Figure 6F**), with 164 upregulated and 141 downregulated genes. A summary of the four sets of comparisons revealed no co-occurring differential genes (**Figure 6E**).

### Enrichment analysis of differentially expressed genes

**Figure 7:**
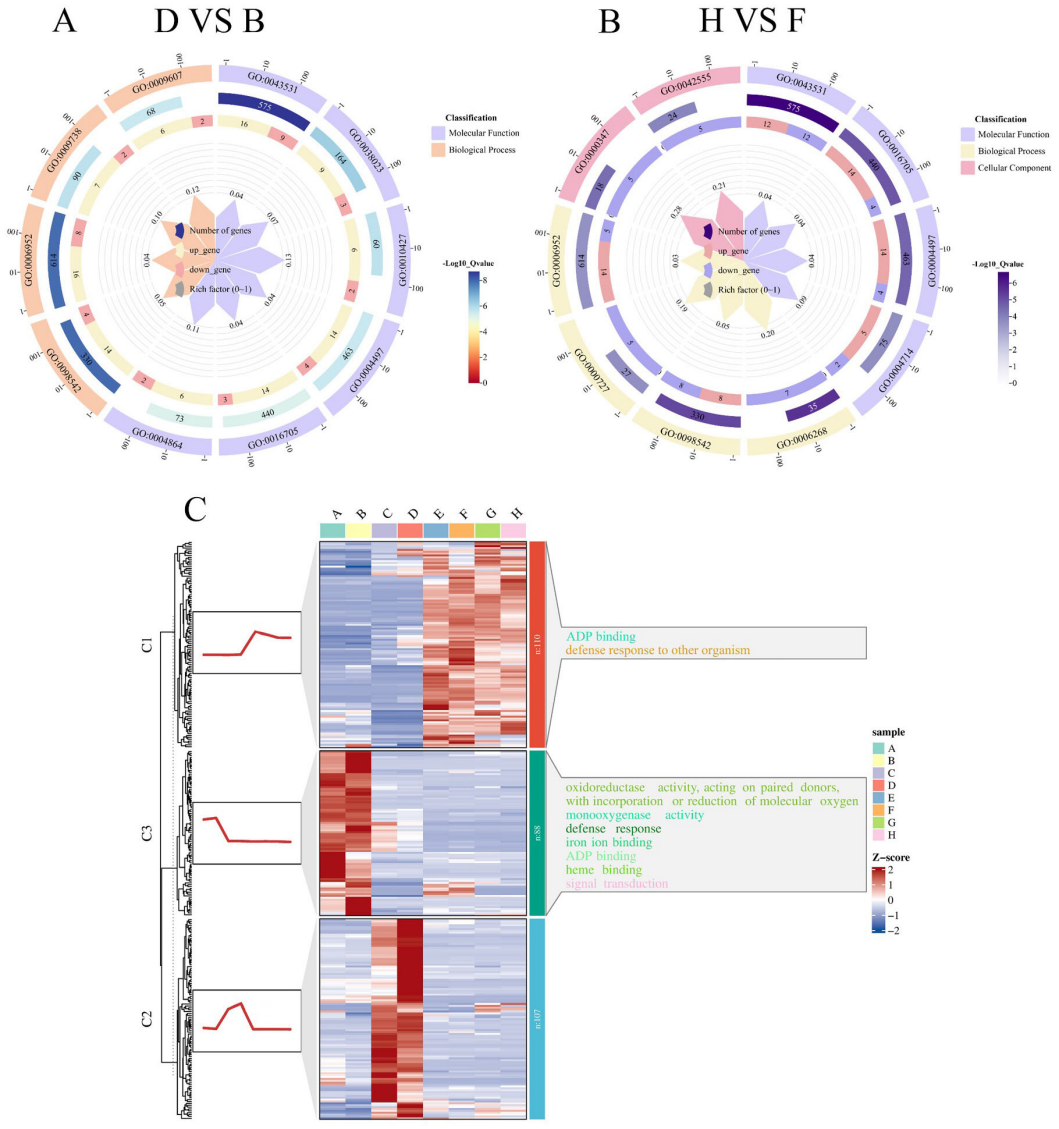
Functional enrichment and clustering trends of DEGs under salt stress in soybean. This figure illustrates the GO enrichment analysis and expression trend clustering of DEGs identified between tolerant and sensitive soybean varieties under salt stress. **(A)** GO enrichment circle plot for the D-VS-B comparison (salt-tolerant R063 vs. salt-sensitive W82 at 36 hours under salt stress). **(B)** GO enrichment circle plot for the H-VS-F comparison (R063 vs. W82 at 48 hours under salt stress). **(C)** K-means clustering analysis of co-expressed DEGs shared across comparisons. Heatmaps on the left depict gene expression trends over time; corresponding enriched GO terms on the right reveal temporal dynamics of stress-response processes.

The identified DEGs were functionally categorized into three main classes: Molecular Function (MF), Biological Process (BP), and Cellular Component (CC). Gene Ontology (GO) enrichment analysis of the D-VS-B comparison revealed the top 10 significantly enriched pathways, as follows (**Figure 7A**): MF includes six pathways: ADP binding (GO:0043531), signaling receptor activity (GO:0038023), abscisic acid binding (GO:0010427), monooxygenase activity (GO:0004497), oxidoreductase activity (GO:0016705), protein phosphatase inhibitor activity(GO:0004864); BP includes four pathways: defense response to other organisms (GO:0098542), defense response (GO:0006952), abscisic acid-activated signaling pathway (GO:0009738), response to biotic stimulus (GO:0009607). Among these, the most significantly enriched pathway was ADP binding (GO:0043531). In a study by Wang et al. (2020) investigating salt-responsive miRNAs and phasiRNAs in soybean roots, GO enrichment analysis of candidate genes also revealed significant enrichment in ADP binding, indicating that this pathway plays a critical role not only in soybean root responses to salt stress but also in soybean seed germination under salt stress.

In the H-VS-F comparison, the top 10 significantly enriched pathways (**Figure 7B**) were categorized as follows: MF includes four pathways: ADP binding (GO:0043531), oxidoreductase activity (GO:0016705), monooxygenase activity (GO:0004497), transmembrane receptor protein tyrosine kinase activity (GO:0004714); BP includes four pathways: DNA unwinding involved in DNA replication (GO:0006268), defense response to other organisms (GO:0098542), double-strand break repair via break-induced replication (GO:0000727), and defense response (GO:0006952); CC includes two pathways: THO complex (GO:0000347), MCM complex (GO:0042555). The most significantly enriched pathway was ADP binding (GO:0043531).

It was observed that at both time points, the D-VS-B and H-VS-F comparisons were enriched in the same pathways, including ADP binding (GO:0043531), monooxygenase activity (GO:0004497), oxidoreductase activity, acting on paired donors, with incorporation or reduction of molecular oxygen (GO:0016705), defense response (GO:0006952), defense response to other organism (GO:0098542). Notably, ADP binding (GO:0043531) was the most significantly enriched pathway in both comparisons. To enhance the understanding of the dynamic expression trends of differential genes, using K-means clustering. The differential genes in each category were subsequently analyzed for GO enrichment. Differential genes shared between the salt-tolerant and salt-sensitive varieties at both time points were grouped into three categories (**Figure 7C**). The first category consisted of 110 DEGs significantly enriched in ADP binding (GO:0043531). The second category, comprising 107 DEGs, was significantly enriched in the assembly of the large subunit precursor of preribosomes. The third category included 88 DEGs, was significantly enriched in the oxidoreductase activity, acting on paired donors, with incorporation or reduction of molecular oxygen (GO:0016705).

**Figure 8:**
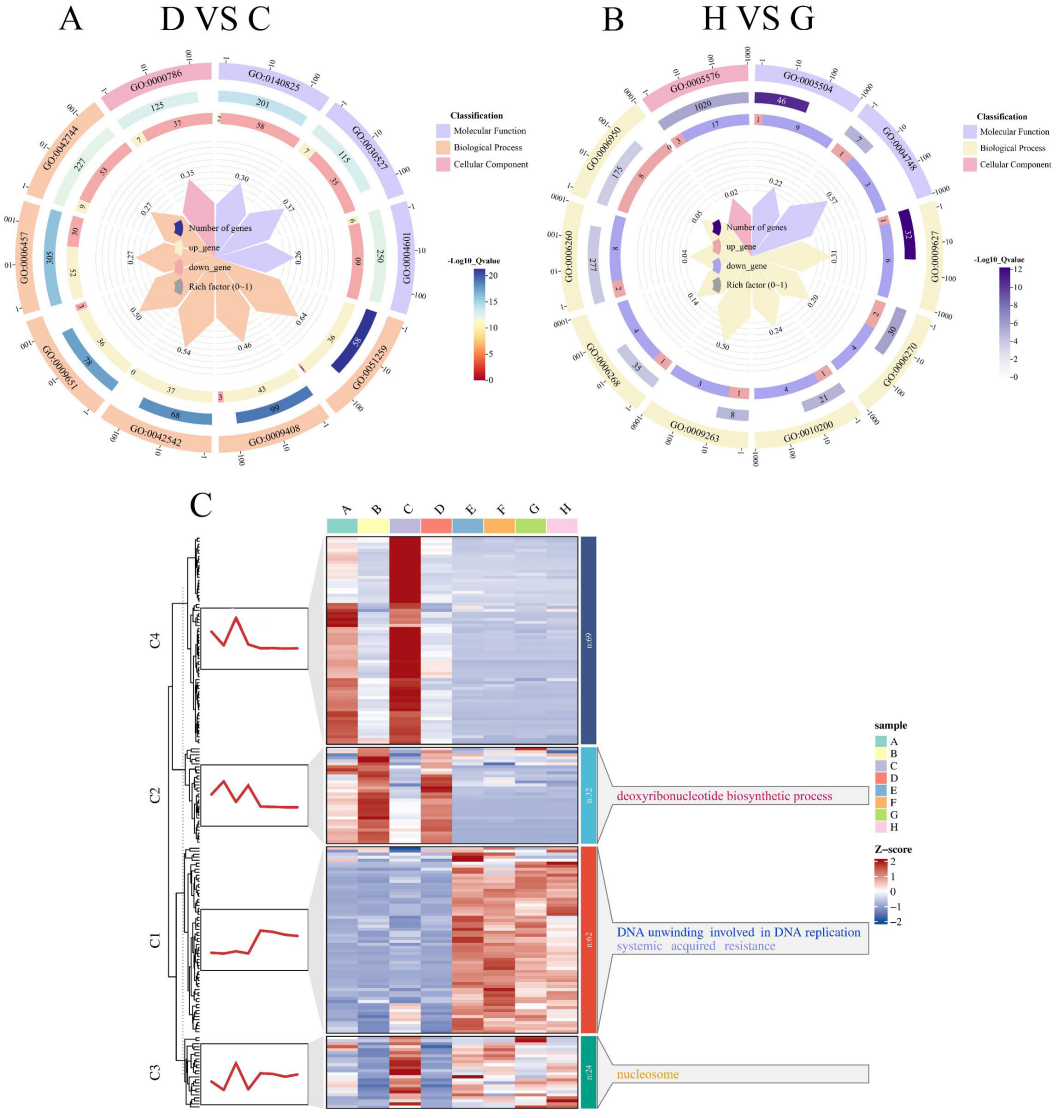
GO functional enrichment and temporal clustering of DEGs in the salt-tolerant soybean genotype R063 under salt stress. This figure displays the functional enrichment results and expression trend dynamics of DEGs in R063 under salt treatment at two time points. **(A)** D-VS-C differentially expressed gene GO enrichment circle plot. **(B)** H-VS-G differentially expressed gene GO enrichment circle plot. **(C)** Clustering trend analysis of differentially expressed genes using the k-means algorithm. The trend plots and the corresponding GO annotations are shown on the left and right sides of the heatmaps, respectively.

In the D-VS-C comparison, the top 10 significantly enriched pathways (**Figure 8A**) were categorized as follows: MF includes three pathways: lactoperoxidase activity (GO:0140825), structural constituent of chromatin (GO:0030527), peroxidase activity (GO:0004601); BP includes seven pathways: protein complex oligomerization (GO:0051259), response to heat (GO:0009408), response to hydrogen peroxide (GO:0042542), response to salt stress (GO:0009651), protein folding (GO:0006457), hydrogen peroxide catabolic process (GO:0042744); CC includes one pathway: nucleosome (GO:0000786). The most significantly enriched pathway was protein complex oligomerization (GO:0051259), while the pathway with the highest number of DEGs was protein folding (GO:0006457). In a study by Gangadhar et al. (2014), which utilized a large-scale yeast functional screening system to explore genes responsive to high-temperature stress in potatoes, it was found that DEGs were significantly enriched in the protein folding pathway. This suggests that the protein folding pathway plays a crucial role not only in plant responses to high-temperature stress but also in salt stress tolerance.

In the H-VS-G comparison, the top 10 significantly enriched pathways (**Figure 8B**) were categorized as follows: MF includes seven pathways: fatty acid binding (GO:0005504); BP includes seven pathways: systemic acquired resistance (GO:0009627), DNA replication initiation (GO:0006270), response to chitin (GO:0010200), deoxyribonucleotide biosynthetic process (GO:0009263), DNA unwinding involved in DNA replication (GO:0006268), DNA replication (GO:0006260), and response to stress (GO:0006950); CC includes one pathways: extracellular region (GO:0005576). The most significantly enriched pathway was systemic acquired resistance (GO:0009627), while the pathway with the highest number of DEGs was the extracellular region (GO:0005576). In a study by Li et al. (2021), which combined transcriptomics and metabolomics analyses on the salt-tolerant plant Nitraria sibirica Pall., GO enrichment analysis of the identified DEGs revealed significant enrichment in the extracellular region pathway. This suggests that this pathway plays a crucial role in salt stress tolerance.

To enhance the understanding of the dynamic expression trends of differential genes, we categorized the differential genes that co-occurred between salt-tolerant varieties and their controls at both time points, using k-means clustering. The differential genes shared between the salt-tolerant varieties and their controls at both time points were divided into four categories (**Figure 8C**). The first category included 62 DEGs, which were significantly enriched in DNA unwinding involved in DNA replication (GO:0006268). The second category comprised 32 DEGs, which were significantly enriched in the deoxyribonucleotide biosynthetic process (GO:0009263). The third category, consisting of 62 DEGs, was nucleosome (GO:0000786). Finally, the fourth category contained 69 DEGs with no significant enrichment in any signaling pathway.

### RT-qPCR analysis of gene expression

**Figure 9:**
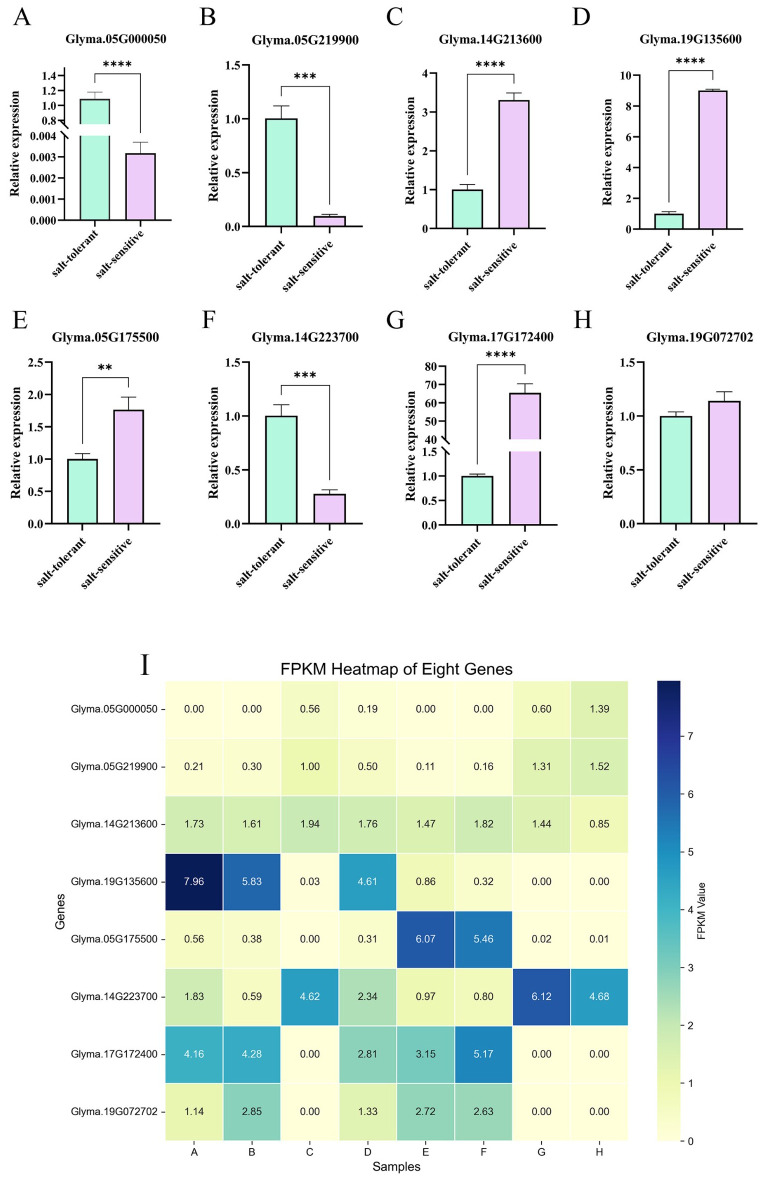
Candidate gene expression validation**(A–H)**. FPKM heatmap of genes**(I)**. The abscissa in the figure is the sample name, and the ordinate is the relative expression. (** means P ≤ 0.01 in T test, *** means P ≤ 0.005in T-test, **** means P ≤ 0.0001 in T-test).

To validate the accuracy of the sequencing results, four candidate genes were randomly selected from the DEGs genes (*Glyma.05G000050* and *Glyma.05G219900*) and two downregulated genes (*Glyma.14G213600* and *Glyma.19G135600*). Similarly, four candidate genes were randomly selected from the DEGs identified in the comparison between Group H and Group F, including two upregulated genes (*Glyma.14G223700* and *Glyma.19G072702*) and two downregulated genes (*Glyma.05G175500* and *Glyma.17G172400*). A heatmap of the FPKM values for these eight candidate genes was generated (**Figure 9**). RT-qPCR analysis was performed on all eight candidate genes. *Glyma.12G051100* was used as the internal reference gene. The relative mRNA expression levels of the target genes were quantified using the Stratagene Mx3005P Real-Time PCR System (Agilent Technologies) and calculated using the 2^-ΔΔCt^ method. The RT-qPCR results were highly consistent with the transcriptome data, indicating a high level of reliability in the RNA-Seq results. Notably, *Glyma.17G172400* exhibited a relatively high expression level in the W82 salt-treated group, whereas Glyma.05G219900 showed a relatively low expression level in the same group. These two genes may serve as promising candidates for future transgenic experiments and functional validation studies.

### WGCNA analysis

WGCNA is a widely employed systems biology methodology for constructing gene co-expression networks, utilizing high-throughput messenger RNA (mRNA) expression data. The underlying assumption of the algorithm is that the gene network follows a scale-free distribution. The process begins by defining a gene co-expression correlation matrix, followed by the computation of an adjacency function to establish the gene network. Next, the algorithm calculates the topological dissimilarity between nodes and constructs a hierarchical clustering tree based on these dissimilarity measures. The branches of the resulting tree correspond to distinct gene modules, with genes within the same module exhibiting high co-expression, while genes from different modules demonstrate low co-expression (Zhu et al., 2021; Zheng et al., 2023; Zhou et al., 2024).

In this experiment, we performed WGCNA on RNA sequencing (RNA-seq) data derived from 24 samples representing two soybean varieties at two-time points under various concentrations of salt treatments during the germination phase. A total of 52,872 differential genes were analyzed. Initially, gene expression data were screened, and the log2-transformed expression values (after adding 1 to each value) were used to filter out genes. To construct a robust co-expression network, we prioritized the top 5,000 genes with the highest median absolute deviation (MAD) across all samples. This selection aimed to focus the WGCNA analysis on genes exhibiting the most pronounced expression variability, a well-established filtering strategy that enhances module detection accuracy and minimizes background noise. WGCNA was then applied to construct a hierarchical clustering tree based on gene expression correlations, dividing the genes into distinct modules. Each module is represented by a different color in the clustering tree. Genes within the same module exhibit similar expression patterns, suggesting they may be functionally related and can be grouped accordingly. In the upper half of the tree, the vertical distance between two nodes (genes) represents their dissimilarity, while the horizontal distance is not relevant.

**Figure 10:**
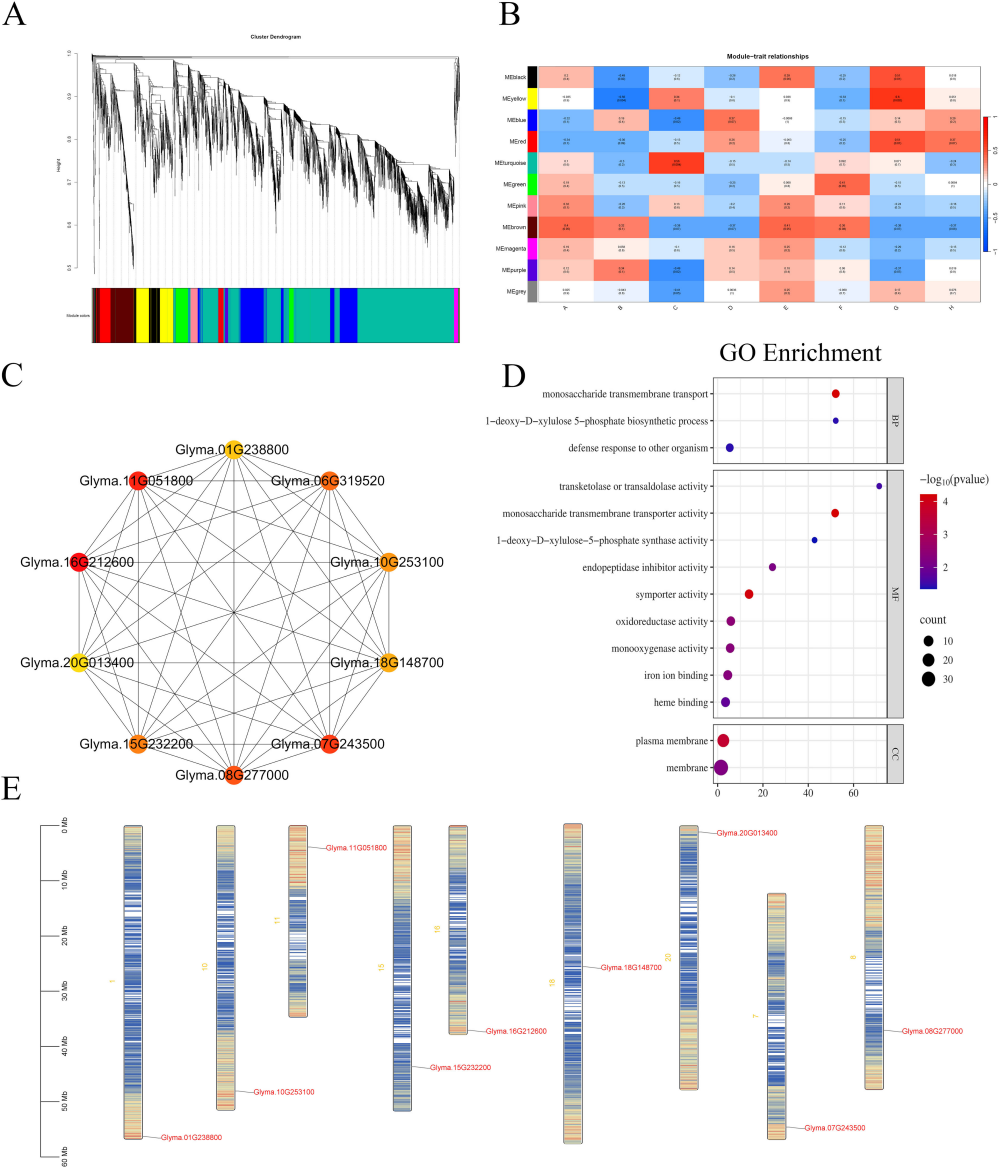
WGCNA. **(A)** Gene Cluster Dendrogram and Module Detection: Displays the hierarchical clustering tree of genes and the detected modules. **(B)** Module-Trait Correlation Heatmap: Illustrates the correlations between modules and sample traits. Each box represents a module-trait relationship, with red indicating a positive correlation and blue indicating a negative correlation. **(C)** Co-expression Network of Top 20 Hub Genes in the Red Module: Visualizes the co-expression relationships among the top 20 hub genes within the red module. **(D)** GO Enrichment Bubble Plot for DEGs in the Red Module: Depicts the GO enrichment analysis results for differentially expressed genes within the red module. **(E)** Chromosomal Localization of the Top 10 Hub Genes: Maps the chromosomal positions of the top 10 hub genes. The x-axis represents different treatments in these figures, and the y-axis represents different modules. Candidate hub genes are highlighted in red. Each node represents a gene, and each edge represents the co-expression correlation between genes.

DEGs were organized into 11 co-expression modules, each assigned a unique color for visualization (**Figure 10A**). The correlation between these modules and DEGs was evaluated based on expression pattern similarity. As depicted in **Figure 10B**, the correlation coefficients and their corresponding P-values between modules and phenotypes were quantified, with larger absolute values indicating stronger relationships (red for positive correlations, blue for negative correlations). Specifically, labels A–D correspond to the 36-hour time point for the W82 control group, W82 salt-treated group, R063 control group, and R063 salt-treated group, respectively, while E–H represent the 48-hour time point for these groups.

Ten hub genes were identified as central nodes in the co-expression network using Cytoscape: *Glyma.01G238800*, *Glyma.11G051800*, *Glyma.06G319520*, *Glyma.16G212600*, *Glyma.10G253100*, *Glyma.20G013400*, *Glyma.18G148700*, *Glyma.15G232200*, *Glyma.07G243500*, and *Glyma.08G277000* (**Figure 10C**). Notably, genes in the red module exhibited distinct expression dynamics: they were upregulated in groups D and H (R063 salt-treated at both time points) but downregulated in groups B and F (W82 salt-treated at both time points).

GO enrichment analysis of red module DEGs (**Figure 10D**) revealed significant overrepresentation in biological processes related to symporter activity (GO:0015293), monosaccharide transmembrane transporter activity (GO:0015145), and monosaccharide transmembrane transport (GO:0015749). Chromosomal localization of the top 10 hub genes is illustrated in **Figure 10E**. Further comparative analysis between the red module and the DB-VS-HF dataset identified 24 overlapping DEGs, among which Glyma.16G212600, Glyma.08G277000, and Glyma.15G232200 were also classified as hub genes. Quantitative fluorescence assays confirmed the expression patterns of these three core genes, aligning with transcriptomic data (**Supplementary Figure 1**).

In this study, Gene Ontology enrichment analysis indicated that *Glyma.08G277000* is involved in “transketolase or transaldolase activity” (GO:0016744), the “1-deoxy-D-xylulose-5-phosphate biosynthetic process” (GO:0052865), and “1-deoxy-D-xylulose-5-phosphate synthase activity” (GO:0008661). These functions are key components of the methylerythritol phosphate (MEP) pathway (Cordoba et al., 2009), which is responsible for the plastid-localized biosynthesis of isoprenoid precursors such as carotenoids and abscisic acid (ABA). The observed salt-induced expression of *Glyma.08G277000* in the tolerant genotype suggests that it may contribute to salt stress adaptation by enhancing the metabolic flux through the MEP pathway. Collectively, these findings imply that *Glyma.08G277000* may play a pivotal role in coordinating metabolic and hormonal responses to salt stress during soybean seed germination.

## Discussion

Salt stress significantly hampers plant development, with seed germination being particularly vulnerable due to the sensitivity of early-stage cellular processes (Shi et al., 2022). In soybeans, successful germination under salinity conditions is a prerequisite for stand establishment and yield stability in saline soils. To survive salt-induced osmotic and oxidative stress, soybeans have evolved multilayered adaptive mechanisms involving ion homeostasis, osmotic adjustment, antioxidative defense, and stress-responsive gene regulation (Leung et al., 2023). Previous studies have identified several key genes that play fundamental roles in the salt tolerance mechanisms of soybean varieties, including *Glyma.02G228100*, *Glyma.03G226000*, *Glyma.03G031000*, *Glyma.03G031400*, *Glyma.04G180300*, *Glyma.04G180400*, *Glyma.05G204600*, *Glyma.08G189600*, *Glyma.13G042200*, and *Glyma.17G17320* (Zeng et al., 2019). In this study, our data revealed that *Glyma.03G031400* and *Glyma.13G042200* were present in both the DB-VS-HF comparison, while *Glyma.04G180400* was only found in the D-VS-B comparison.

Notably, we observed a sharp reduction in the number of DEGs in the salt-tolerant R063 between 36 hours and 48 hours post salt treatment, from 6,591 to only 438. This rapid contraction in transcriptomic activity may suggest that R063 possesses a fast-acting regulatory mechanism, enabling swift stress perception and subsequent stabilization of gene expression. Based on these dynamics, we hypothesize that the 36-hour time point may represent an “activation phase” in which the plant initiates extensive transcriptional reprogramming in response to salt stress, while the 48-hour point may correspond to a “dynamic equilibrium phase” reflecting a return toward homeostasis. We emphasize that these terms represent our interpretation of the observed gene expression patterns, rather than definitive physiological states. This proposed framework aligns with models of rapid adaptation and transcriptional stabilization that have been previously associated with abiotic stress tolerance.

Interestingly, when comparing the tolerant and sensitive genotypes under salt stress (D-VS-B and H-VS-F), the DEG numbers were surprisingly low (656 and 826, respectively), which implies that R063’s advantage may not arise from an amplified inducible response, but rather from pre-configured gene expression or post-transcriptional regulation. This observation supports the notion of a ‘primed’ or ‘readiness’ state in salt-tolerant seeds, a concept previously reported in priming studies (Johnson and Puthur, 2021).

The physiological responses of the salt-tolerant genotype R063 under stress conditions were consistent with its transcriptomic profile. Compared to the sensitive variety, R063 showed significantly elevated levels of SOD and POD activities, while malondialdehyde MDA content was markedly lower. These results suggest that R063 is better equipped to neutralize reactive ROS and minimize lipid peroxidation in cell membranes—key mechanisms for surviving salinity stress. Interestingly, the physiological data align with the transcriptomic findings. For instance, GO terms such as “oxidoreductase activity” and “defense response” were enriched among DEGs, directly linking to antioxidant functions.

Through statistical analysis of the GO enrichment results for multiple groups of DEGs, we identified ten key pathways significantly associated with soybean tolerance to salt stress. These pathways include ADP binding (GO:0043531), monooxygenase activity (GO:0004497), oxidoreductase activity, acting on paired donors, with incorporation or reduction of molecular oxygen (GO:0016705), defense response (GO:0006952), defense response to other organism (GO:0098542), oxidoreductase activity, acting on paired donors, with incorporation or reduction of molecular oxygen (GO:0016705), DNA unwinding involved in DNA replication (GO:0006268), deoxyribonucleotide biosynthetic process (GO:0009263), nucleosome (GO:0000786). These pathways play critical roles in soybean’s resistance to salt stress, and our findings are consistent with previous reports. For instance, Feng et al. (2017) conducted a GO enrichment analysis on DEGs from wild emmer wheat under salt stress, identifying significant enrichment in protein phosphorylation, ATP binding, and ADP binding pathways. Similarly, Li et al. (2019) analyzed DEGs in transgenic poplars under salt stress and found a prominent expression of monooxygenase and oxidoreductase activities. The enrichment of GO terms such as “ADP binding” among DEGs under salt stress suggests a mechanistic connection between energy metabolism and stress adaptation. ADP-binding proteins, including H^+^-ATPases and kinases, are central to maintaining energy-demanding processes critical for salt tolerance (Guo et al., 2022). This analysis bridges broad GO enrichments to functional insights, advancing our understanding of salinity resilience at the molecular level.

WGCNA is a powerful tool for identifying gene sets that exhibit highly coordinated variation and for mining candidate genes through the interconnectivity and associations between gene sets and phenotypes. WGCNA has been widely applied across various species to identify candidate genes regulating target traits (Lu et al., 2019; Chen et al., 2020; Ma et al., 2021). In order to identify hub genes associated with salt tolerance in soybeans, we performed WGCNA using FPKM values of DEGs. The results revealed 11 distinct modules (**Figure 10B**). In the red module, we identified 10 core genes, among which *Glyma.18G148700* and *Glyma.08G277000* were found to be upregulated under acidity stress and downregulated under drought stress (Jahan et al., 2019). These genes were also expressed following salt stress treatment. A comparison between the differentially expressed genes obtained from the DB-VS-HF dataset and those identified in the red module revealed 24 overlapping genes, including *Glyma.16G212600*, *Glyma.08G277000*, and *Glyma.15G232200*, all of which appeared among the top 10 core genes. At present, this study has not conducted further validation of these core genes. Future work will involve more in-depth functional analysis of these core genes, as well as a comprehensive investigation into the signaling pathways that were significantly enriched with differential genes. Based on GO annotations and prior literature, we propose the following hypotheses: *Glyma.08G277000* encodes a transketolase involved in the methylerythritol phosphate (MEP) pathway, which regulates plastidial isoprenoid synthesis, including abscisic acid (ABA)—a critical hormone for salt response. Its upregulation may enhance ABA production during germination.

This study integrated RNA-seq technology with WGCNA to systematically investigate the transcriptomic response mechanisms of soybean under salt stress, providing molecular insights into the genetic basis of salt tolerance in soybean. The results revealed that salt stress enhances salt tolerance in soybean by regulating multiple key genes and signaling pathways. GO enrichment analysis indicated that DEGs were significantly enriched in the ADP-binding pathway. Furthermore, WGCNA analysis identified three hub genes closely associated with salt stress responses: *Glyma.16G212600*, *Glyma.08G277000*, and *Glyma.15G232200*, which serve as molecular targets for future studies on improving soybean salt tolerance. This study offers testable molecular targets for improving salt resilience in early-stage soybean development.

## Conclusion

This study comprehensively examined salt stress effects on soybean seed germination using physiological assays and transcriptomic profiling. The findings revealed that SOD plays a crucial regulatory role in salt stress tolerance during soybean germination. In addition, two potential salt-tolerant genes, *Glyma.03G031400* and *Glyma.13G04220*, were identified and verified in this study, building upon previous research data. Through a combined analysis of transcriptome sequencing and WGCNA, we identified key genes associated with salt tolerance during soybean germination. GO enrichment analysis of differentially expressed genes revealed that the ADP binding (GO:0043531) signaling pathway is integral to salt stress tolerance during soybean germination. Further WGCNA analysis identified hub genes, all of which play significant roles in regulating salt stress tolerance during soybean germination. Among these, *Glyma.16G212600* was found to be the most critical gene. This study provides valuable theoretical insights and practical experience for the identification of genes related to salt stress tolerance during soybean germination, offering a robust genetic resource for future research in this area.

## Data Availability

The data presented in the study are deposited in the NCBI SRA repository, accession number PRJNA 1252398.
