# Supplementary material for: Using WGCNA and transcriptome profiling to identify hub genes for salt stress tolerance in germinating soybean seeds
Source: Front Plant Sci. 2025 Aug 8;16:1569565. doi: 10.3389/fpls.2025.1569565 (PMC12370657; doi:10.3389/fpls.2025.1569565)
Supplement: Supplementary file 1 [file DataSheet1.zip › Supplemental table2.pptx]

## Slide 1
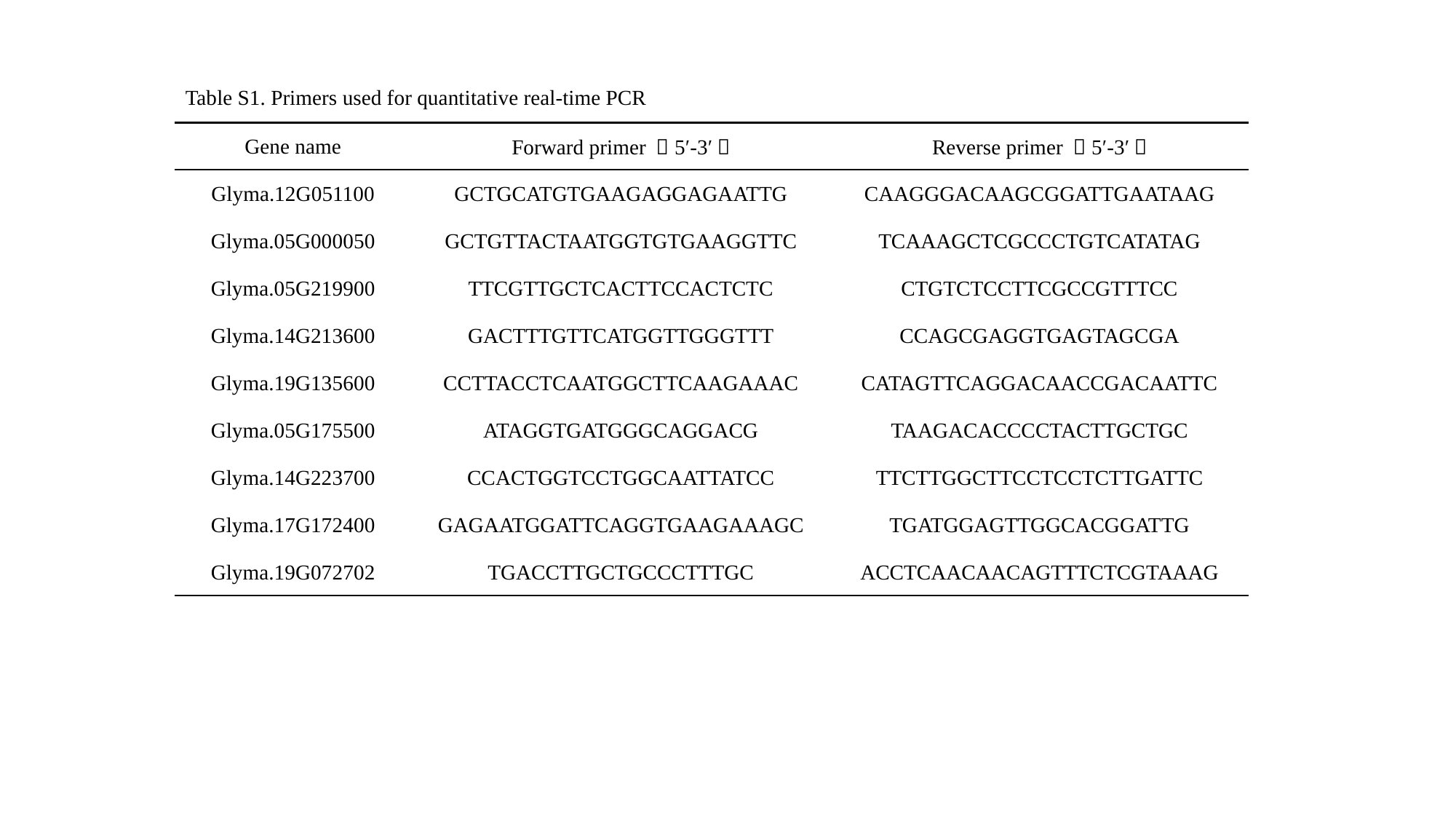

Table S1. Primers used for quantitative real-time PCR
| Gene name | Forward primer （5′-3′） | Reverse primer （5′-3′） |
| --- | --- | --- |
| Glyma.12G051100 | GCTGCATGTGAAGAGGAGAATTG | CAAGGGACAAGCGGATTGAATAAG |
| Glyma.05G000050 | GCTGTTACTAATGGTGTGAAGGTTC | TCAAAGCTCGCCCTGTCATATAG |
| Glyma.05G219900 | TTCGTTGCTCACTTCCACTCTC | CTGTCTCCTTCGCCGTTTCC |
| Glyma.14G213600 | GACTTTGTTCATGGTTGGGTTT | CCAGCGAGGTGAGTAGCGA |
| Glyma.19G135600 | CCTTACCTCAATGGCTTCAAGAAAC | CATAGTTCAGGACAACCGACAATTC |
| Glyma.05G175500 | ATAGGTGATGGGCAGGACG | TAAGACACCCCTACTTGCTGC |
| Glyma.14G223700 | CCACTGGTCCTGGCAATTATCC | TTCTTGGCTTCCTCCTCTTGATTC |
| Glyma.17G172400 | GAGAATGGATTCAGGTGAAGAAAGC | TGATGGAGTTGGCACGGATTG |
| Glyma.19G072702 | TGACCTTGCTGCCCTTTGC | ACCTCAACAACAGTTTCTCGTAAAG |

## Slide 2
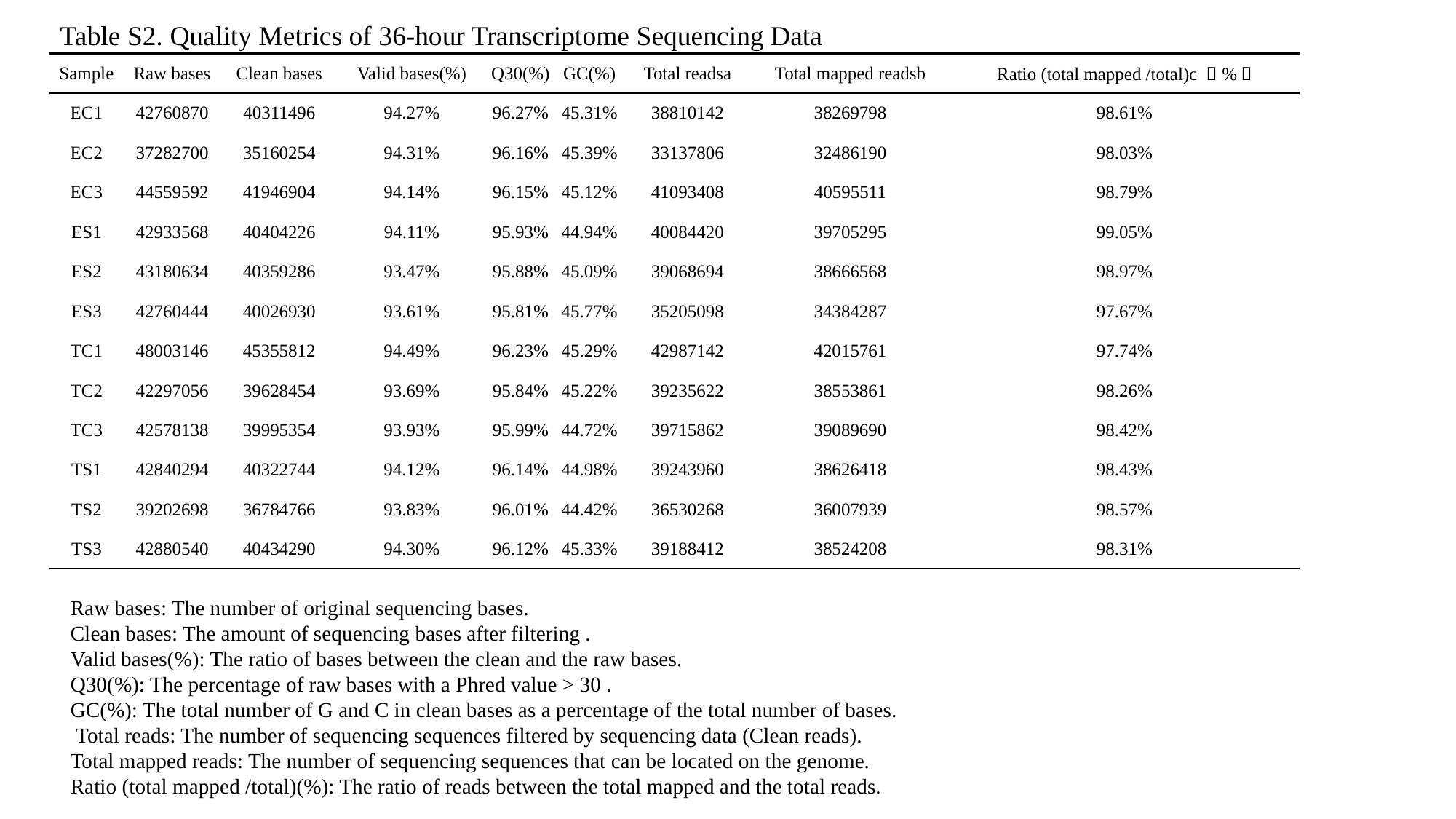

Table S2. Quality Metrics of 36-hour Transcriptome Sequencing Data
| Sample | Raw bases | Clean bases | Valid bases(%) | Q30(%) | GC(%) | Total readsa | Total mapped readsb | Ratio (total mapped /total)c （%） |
| --- | --- | --- | --- | --- | --- | --- | --- | --- |
| EC1 | 42760870 | 40311496 | 94.27% | 96.27% | 45.31% | 38810142 | 38269798 | 98.61% |
| EC2 | 37282700 | 35160254 | 94.31% | 96.16% | 45.39% | 33137806 | 32486190 | 98.03% |
| EC3 | 44559592 | 41946904 | 94.14% | 96.15% | 45.12% | 41093408 | 40595511 | 98.79% |
| ES1 | 42933568 | 40404226 | 94.11% | 95.93% | 44.94% | 40084420 | 39705295 | 99.05% |
| ES2 | 43180634 | 40359286 | 93.47% | 95.88% | 45.09% | 39068694 | 38666568 | 98.97% |
| ES3 | 42760444 | 40026930 | 93.61% | 95.81% | 45.77% | 35205098 | 34384287 | 97.67% |
| TC1 | 48003146 | 45355812 | 94.49% | 96.23% | 45.29% | 42987142 | 42015761 | 97.74% |
| TC2 | 42297056 | 39628454 | 93.69% | 95.84% | 45.22% | 39235622 | 38553861 | 98.26% |
| TC3 | 42578138 | 39995354 | 93.93% | 95.99% | 44.72% | 39715862 | 39089690 | 98.42% |
| TS1 | 42840294 | 40322744 | 94.12% | 96.14% | 44.98% | 39243960 | 38626418 | 98.43% |
| TS2 | 39202698 | 36784766 | 93.83% | 96.01% | 44.42% | 36530268 | 36007939 | 98.57% |
| TS3 | 42880540 | 40434290 | 94.30% | 96.12% | 45.33% | 39188412 | 38524208 | 98.31% |
Raw bases: The number of original sequencing bases.
Clean bases: The amount of sequencing bases after filtering .
Valid bases(%): The ratio of bases between the clean and the raw bases.
Q30(%): The percentage of raw bases with a Phred value > 30 .
GC(%): The total number of G and C in clean bases as a percentage of the total number of bases.
 Total reads: The number of sequencing sequences filtered by sequencing data (Clean reads).
Total mapped reads: The number of sequencing sequences that can be located on the genome.
Ratio (total mapped /total)(%): The ratio of reads between the total mapped and the total reads.

## Slide 3
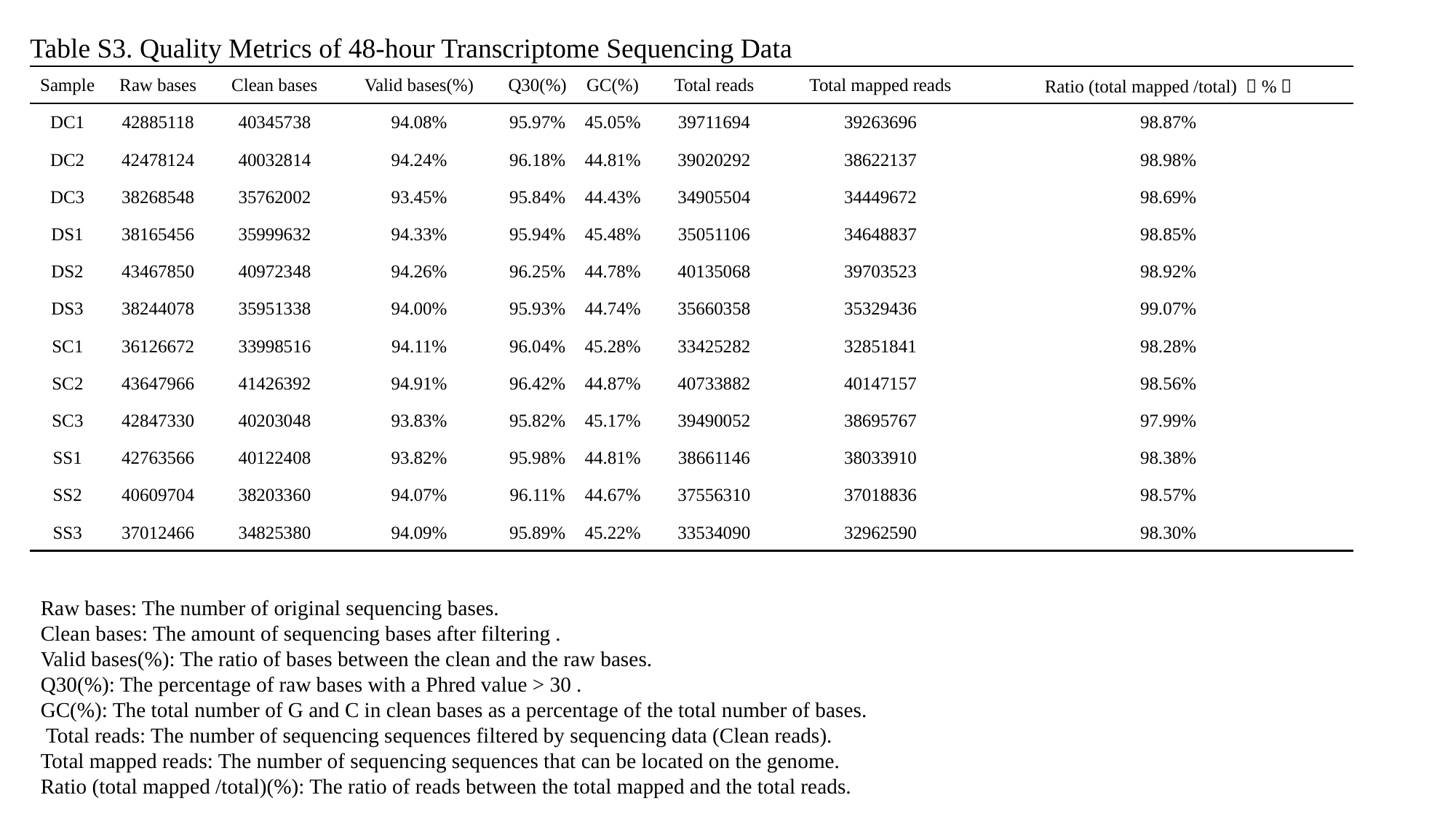

Table S3. Quality Metrics of 48-hour Transcriptome Sequencing Data
| Sample | Raw bases | Clean bases | Valid bases(%) | Q30(%) | GC(%) | Total reads | Total mapped reads | Ratio (total mapped /total) （%） |
| --- | --- | --- | --- | --- | --- | --- | --- | --- |
| DC1 | 42885118 | 40345738 | 94.08% | 95.97% | 45.05% | 39711694 | 39263696 | 98.87% |
| DC2 | 42478124 | 40032814 | 94.24% | 96.18% | 44.81% | 39020292 | 38622137 | 98.98% |
| DC3 | 38268548 | 35762002 | 93.45% | 95.84% | 44.43% | 34905504 | 34449672 | 98.69% |
| DS1 | 38165456 | 35999632 | 94.33% | 95.94% | 45.48% | 35051106 | 34648837 | 98.85% |
| DS2 | 43467850 | 40972348 | 94.26% | 96.25% | 44.78% | 40135068 | 39703523 | 98.92% |
| DS3 | 38244078 | 35951338 | 94.00% | 95.93% | 44.74% | 35660358 | 35329436 | 99.07% |
| SC1 | 36126672 | 33998516 | 94.11% | 96.04% | 45.28% | 33425282 | 32851841 | 98.28% |
| SC2 | 43647966 | 41426392 | 94.91% | 96.42% | 44.87% | 40733882 | 40147157 | 98.56% |
| SC3 | 42847330 | 40203048 | 93.83% | 95.82% | 45.17% | 39490052 | 38695767 | 97.99% |
| SS1 | 42763566 | 40122408 | 93.82% | 95.98% | 44.81% | 38661146 | 38033910 | 98.38% |
| SS2 | 40609704 | 38203360 | 94.07% | 96.11% | 44.67% | 37556310 | 37018836 | 98.57% |
| SS3 | 37012466 | 34825380 | 94.09% | 95.89% | 45.22% | 33534090 | 32962590 | 98.30% |
Raw bases: The number of original sequencing bases.
Clean bases: The amount of sequencing bases after filtering .
Valid bases(%): The ratio of bases between the clean and the raw bases.
Q30(%): The percentage of raw bases with a Phred value > 30 .
GC(%): The total number of G and C in clean bases as a percentage of the total number of bases.
 Total reads: The number of sequencing sequences filtered by sequencing data (Clean reads).
Total mapped reads: The number of sequencing sequences that can be located on the genome.
Ratio (total mapped /total)(%): The ratio of reads between the total mapped and the total reads.

## Slide 4
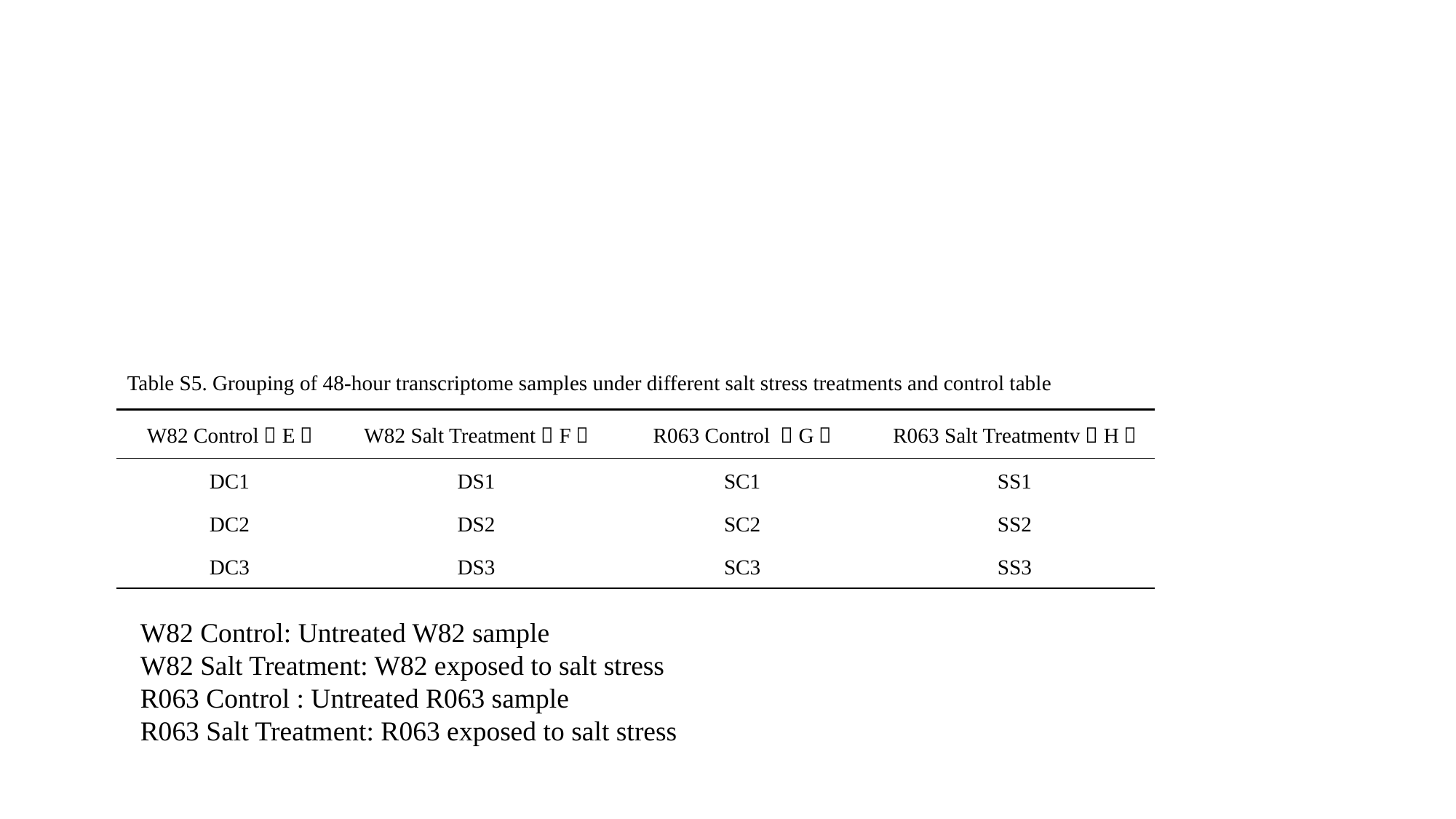

Table S5. Grouping of 48-hour transcriptome samples under different salt stress treatments and control table
| W82 Control（E） | W82 Salt Treatment（F） | R063 Control （G） | R063 Salt Treatmentv（H） |
| --- | --- | --- | --- |
| DC1 | DS1 | SC1 | SS1 |
| DC2 | DS2 | SC2 | SS2 |
| DC3 | DS3 | SC3 | SS3 |
W82 Control: Untreated W82 sample
W82 Salt Treatment: W82 exposed to salt stress
R063 Control : Untreated R063 sample
R063 Salt Treatment: R063 exposed to salt stress

## Slide 5
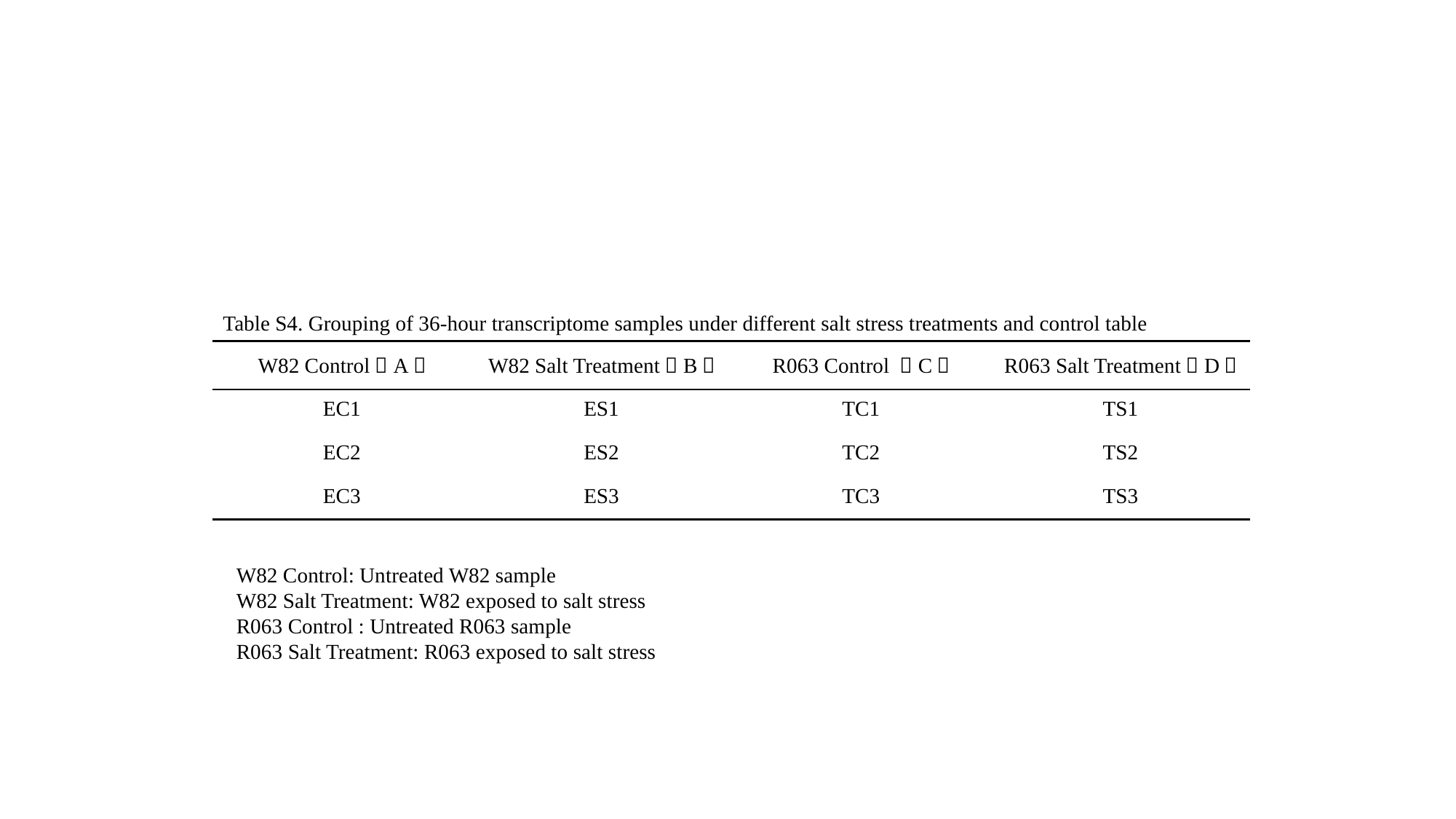

Table S4. Grouping of 36-hour transcriptome samples under different salt stress treatments and control table
| W82 Control（A） | W82 Salt Treatment（B） | R063 Control （C） | R063 Salt Treatment（D） |
| --- | --- | --- | --- |
| EC1 | ES1 | TC1 | TS1 |
| EC2 | ES2 | TC2 | TS2 |
| EC3 | ES3 | TC3 | TS3 |
W82 Control: Untreated W82 sample
W82 Salt Treatment: W82 exposed to salt stress
R063 Control : Untreated R063 sample
R063 Salt Treatment: R063 exposed to salt stress

## Slide 6
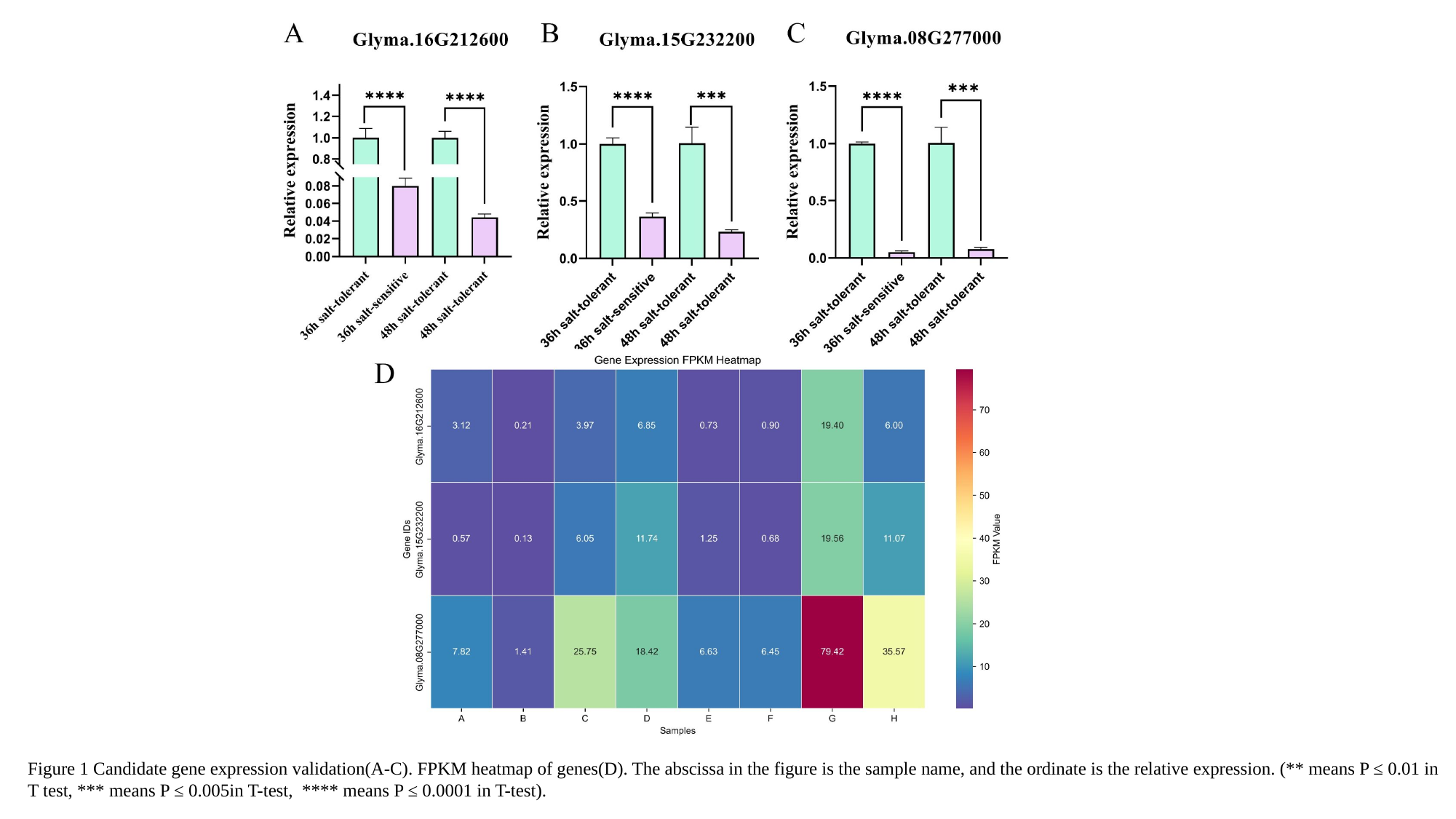

Figure 1 Candidate gene expression validation(A-C). FPKM heatmap of genes(D). The abscissa in the figure is the sample name, and the ordinate is the relative expression. (** means P ≤ 0.01 in T test, *** means P ≤ 0.005in T-test, **** means P ≤ 0.0001 in T-test).

## Slide 7
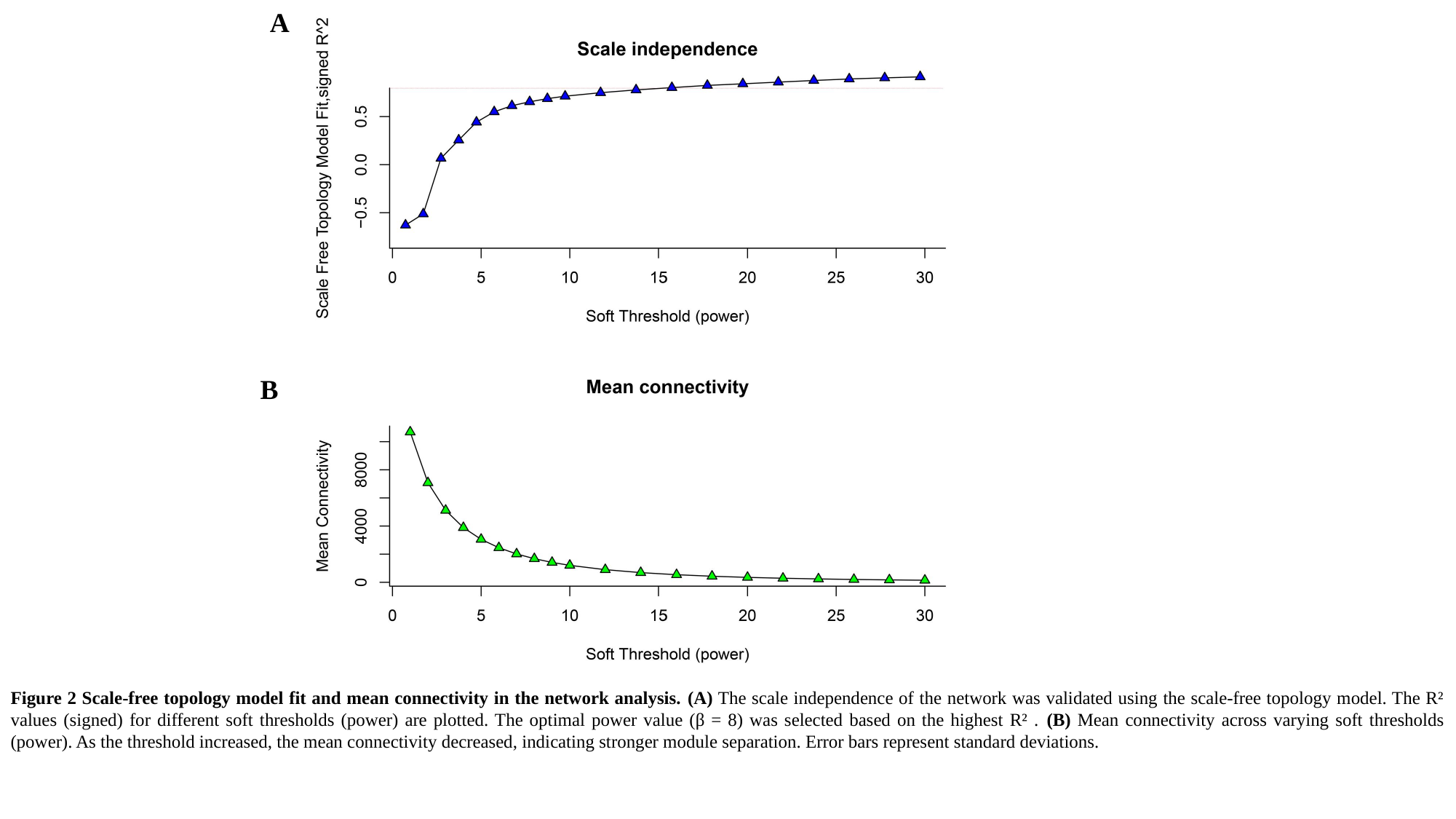

A
B
Figure 2 Scale-free topology model fit and mean connectivity in the network analysis. (A) The scale independence of the network was validated using the scale-free topology model. The R² values (signed) for different soft thresholds (power) are plotted. The optimal power value (β = 8) was selected based on the highest R² . (B) Mean connectivity across varying soft thresholds (power). As the threshold increased, the mean connectivity decreased, indicating stronger module separation. Error bars represent standard deviations.
